# Quantitative Imaging features Improve Discrimination of Malignancy in Pulmonary nodules

**DOI:** 10.1038/s41598-019-44562-z

**Published:** 2019-06-12

**Authors:** Yoganand Balagurunathan, Matthew B. Schabath, Hua Wang, Ying Liu, Robert J. Gillies

**Affiliations:** 1 Quantitative Sciences- Department of Bioinformatics & Biostatistics, H. Lee. Moffitt Cancer Center, Tampa, FL USA; 2 Department of Radiology, H. Lee. Moffitt Cancer Center, Tampa, FL USA; 3 Genitourinary Oncology, H. Lee. Moffitt Cancer Center, Tampa, FL USA; 4 Cancer Epidemiology, H. Lee. Moffitt Cancer Center, Tampa, FL USA; 5 Cancer Physiology, H. Lee. Moffitt Cancer Center, Tampa, FL USA; 6 Department of Radiology, Tianjin Medical University Cancer Institute and Hospital, Tianjin, China

**Keywords:** Cancer imaging, Predictive markers

## Abstract

Pulmonary nodules are frequently detected radiological abnormalities in lung cancer screening. Nodules of the highest- and lowest-risk for cancer are often easily diagnosed by a trained radiologist there is still a high rate of indeterminate pulmonary nodules (IPN) of unknown risk. Here, we test the hypothesis that computer extracted quantitative features (“radiomics”) can provide improved risk-assessment in the diagnostic setting. Nodules were segmented in 3D and 219 quantitative features are extracted from these volumes. Using these features novel malignancy risk predictors are formed with various stratifications based on size, shape and texture feature categories. We used images and data from the National Lung Screening Trial (NLST), curated a subset of 479 participants (244 for training and 235 for testing) that included incident lung cancers and nodule-positive controls. After removing redundant and non-reproducible features, optimal linear classifiers with area under the receiver operator characteristics (AUROC) curves were used with an exhaustive search approach to find a discriminant set of image features, which were validated in an independent test dataset. We identified several strong predictive models, using size and shape features the highest AUROC was 0.80. Using non-size based features the highest AUROC was 0.85. Combining features from all the categories, the highest AUROC were 0.83.

## Introduction

Lung cancer is the largest cause of cancer death in the U.S. and globally^1^. The 5-year survival for patients diagnosed with a non-small cell lung carcinoma remains dismal at 21%, largely attributed to lack of early detection^2^. The 5-year survival for early stage IA patients is 49%. In order to improve methods for early detection; the NLST was a randomized clinical trial to compare low-dose computed tomography (LDCT) to standard chest X-ray (CXR) for three annual screens in high-risk current or former smokers between 55 to 74 years of age with a smoking history of at least 30 pack-years. The NLST enrolled 53,439 participants, of which 26,715 were randomized to the LDCT arm^3^. According to the NLST protocol, “positive screens” were defined as identifying ≥1 non-calcified nodules or masses measuring ≥4 mm in axial diameter or, less commonly, other abnormalities such as adenopathy or pleural effusion. Nodule-positive screens were defined in the setting of abnormalities on baseline screens or abnormalities on incidence screens that were new, stable, or that evolved. The latter was demonstrated by an increase in nodule size, consistency, or other characteristic potentially related to lung cancer. “Negative screens”, which were not included in this analysis, were defined as scans with no confirmed or minor abnormalities not suspicious for lung cancer, or significant abnormalities not suspicious for lung cancer^4^. After a median follow up of 6.4 years, the NLST showed a reduction in overall mortality up to 20% among individuals in the LDCT arm compared to those screened with CXR^4^. These results of reduced mortality in the LDCT arm led recent US Preventive Services Task Force recommendations of LDCT screening for high-risk individuals, despite concern for a high false discovery rate^5^.

Despite the success of the NSLT and evolving national clinical guidelines for the management of pulmonary nodules^6,7^, over detection remains a major concern as it can results in additional patient anxiety, work-up and treatment incurring additional costs and morbidity^8^. In the NLST, among the positive LDCT screens (a nodule 4 mm or larger or a finding suspicious for lung cancer), 96.4% were false positives. Furthermore, in the NLST over 18% of all lung cancers detected by LDCT have been reported to be indolent, diagnosed by secondary histological validation^9^.

One of the challenges in lung cancer screening with LDCT is the high rate of detection of indeterminate pulmonary nodules (IPNs). Although the definitions of IPNs are clinically controversial, they are generally non-calcified nodules (NCN) of intermediate size from 4 mm to 12 mm diameter. The most common method for diagnosing malignancy is evidence of growth over two or more subsequent screen, nodule doubling times (based on diameter or volume) has been shown to predict cancer status^10,11^. Recently risk models based on nodule characteristics, anatomical location, lifestyle factors has been shown to be predictors of malignancy^12–14^. Development of improved discrimination methods could assign risk and inform the need and timing for follow up scans for definitive diagnosis. The clinical recommendation typically follows the consensus expert opinion and recently published studies have provided some guidelines in the management of IPNs^15–17^. The challenge in managing IPN’s coupled with slow adoption of quantitative metrics beyond size would potentially delay or possibly avoid further invasive clinical follow-up such as biopsy.

**Figure 1:**
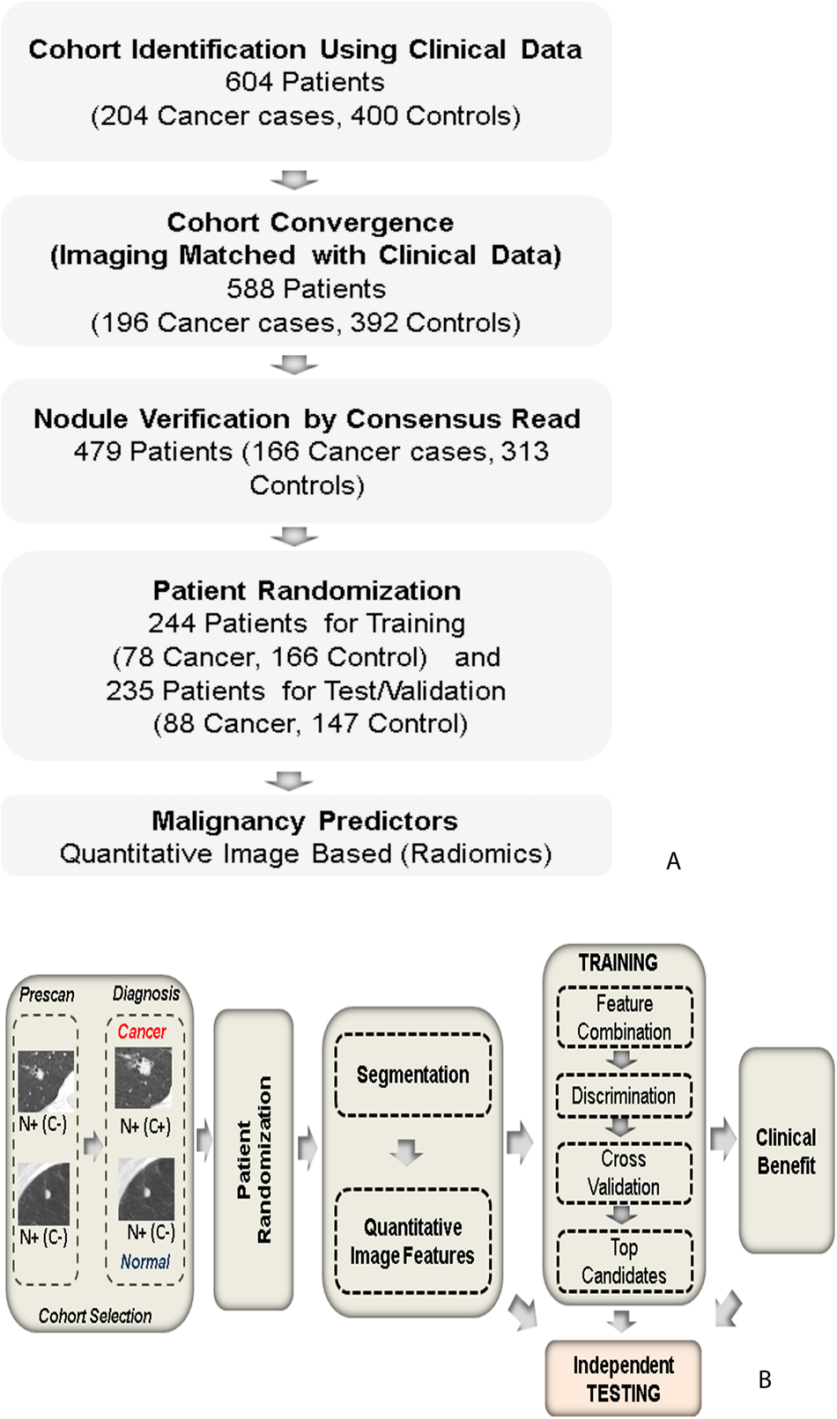
Systematic process followed in the study. (**A**) Cohort identification flowchart, (**B**) Quantitative imaging (Radiomic) based predictor work flow, where N+/C+ indicate presence of a nodule that are confirmed to be malignant, while N+/C− indicate presence of a nodule but remains benign, following NLST inclusion criteria.

In this study we performed a *post hoc* analysis using images and patient data from the NLST with an objective to evaluate the use of quantitative imaging features (radiomics) as markers to predict cancer status of lung nodules. We identified parsimonious set of features to build predictors on a training set of patients and independently tested in a validation cohort of patients. The study also investigates performance of quantitative image based predictors from various descriptor categories and sizes. Figure 1 illustrates the approach of this study.

## Results

**Figure 2:**
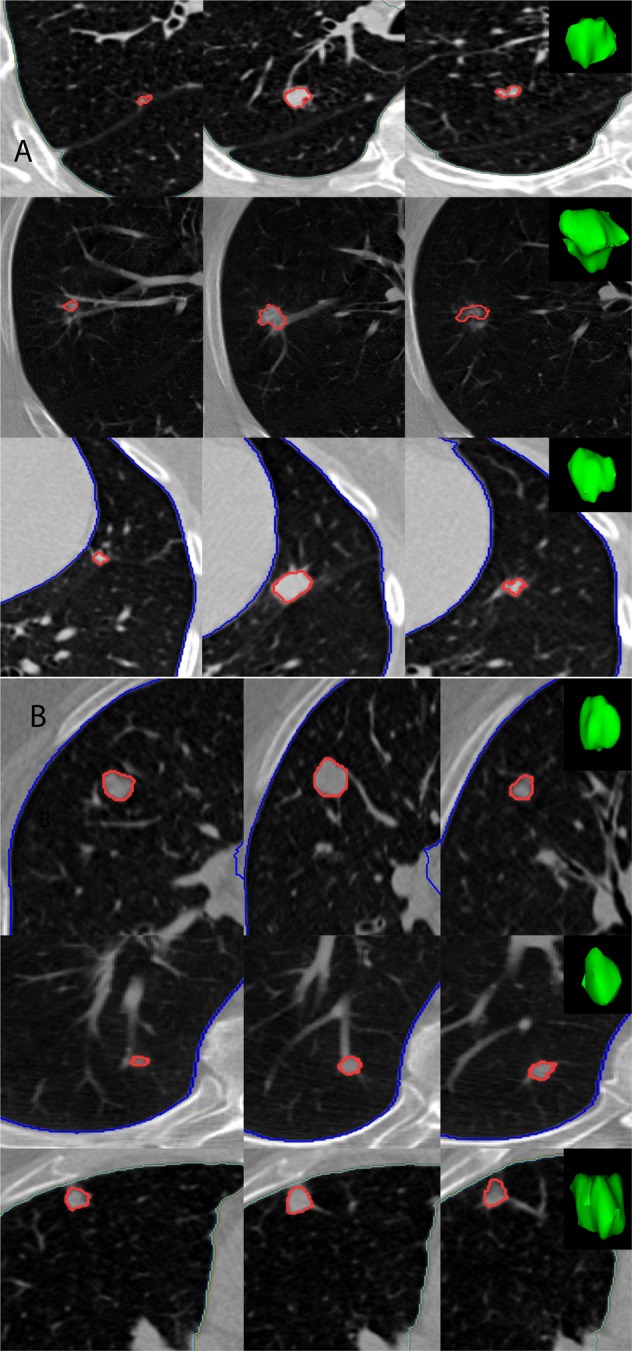
Representative 2D slices of patient LDCT with 3D rendering of the pulmonary nodule of interest, where each row corresponds to a patient (**A**) Patient with confirmed malignant nodule (**B**) Patients with benign nodules, as reported by NLST.

In this study 479 nodule-positive participants were randomly divided into training (78 cases and 166 controls) and test sets (88 cases and 147 controls), see Tables 1 and 2. The mean age at randomization for the participants in training set was 63.9 (standard deviation (σ) = 5.2) years with a mean pack-years smoking history of 63.8 (σ = 25.2). For the test set, the mean age was 63.3 (σ = 4.9) years with a mean smoking history of 61.8 (σ = 22.2) pack-years. Participants were predominantly Caucasian with a sex ratio (male to female) of 1.12 in the training set and 1.67 in the test set. The nodules diagnosed as lung cancer were larger in size compared to nodule positive controls and the size distribution is shown in Supplemental Fig. SF3. Figure 2 shows six representative subjects’ pulmonary nodules with known malignancy status. Visually, other than size there are no distinct differences.

**Table 1:** Demographic information for the Train and test cohort used in the study (for more details referrer to Supplemental Table S1).

	Category	Training			Testing				P-value
		Overall	Lung Cancer Cases	Nodule + Controls	P-value	Overall	Lung Cancer Cases	Nodule + Controls	
1	Demographic Patients	244	78	166		235	88	147	
2	**Nodule Size Distribution**								
	Indeterminate Size Range (longest diameter): (4 mm to 12 mm)	171	32	139		162	31	131	
	Larger Size Range (longest diameter): (>12 mm to 30 mm)	56	38	18		54	46	8	
	Others (>30 mm)	17				19			
3	Age at Diagnosis (Yrs) (Mean (STD), Median)	63.9 (5.2), 64	64.4 (5.4), 64.5	63.6 (5.1),63	***0.28***	63.3 (4.9), 63	63.3 (4.7), 63.5	63.2 (4.9),63	***0.87***
4	Gender (Male:Female)	1.12	1	1.18	***0.58***	1.67	1.44	1.82	***0.41***
5	Male, N	129	39	90		147	52	95	
6	Female, N	115	39	76		88	36	52	
	Ratio (Male/Female)	1.122	1	1.184		1.671	1.44	1.826	
7	Pack Years smoking (Mean (STD), Median)	63.8 (25.2), 57	62.8 (25.7), 54.5	64.2 (24.9), 58	***0.67***	61.8 (22.1), 55.5	62.5 (22.2), 61.5	61.4 (22.1), 54	***0.71***
8	Race(White/Black/Native/Asian/Others), N	238/3/0/3/0	77/1/0/0/0	161/2/0/3/0	***0.67***	221/7/3/0/4	82/5/0/0/1	139/2/3/0/3	***0.77***
	Race(White/Black/Native/Asian/Others), Percent (%)	97/1.23/0/1.23/0	98.7%/1.28/0/0/0	96.98/1.21/0/1.81/0		94/2.97/1.28/0/1.7	93.18/5.68/0/0/1.14	94.56/1.36/2.04/0/2.04	
9	Smoke status (current/former), N (ratio)	130/114 (1.140)	35/43 (0.813)	95/71 (1.338)	***0.076***	125/110 (1.136)	50/38 (1.315)	75/72 (1.042)	***0.42***

The p-values were computed using Students t test for continues variable and Fishers exact test for categorical data.

**Table 2:** Qualitative comparison of CT reconstruction kernel across scanner manufacturers.

Manufacturer	Followed Choice	Alternative Choice
GE	Standard	Lung/Bone
SIEMENS	B30f	B50f
PHILIPS	C	D
TOSHIBA	FC01/FC10	FC30/FC51

First, we used all 129 repeatable features without categorization to find the best 4 feature combinations by exhaustively searching the feature space. Table 3 presents three sets of 4-dimensional features that were significantly different between the lung cancer cases and nodule + controls (Supplemental Table S8). The top radiomic candidates were from multiple categories, including size, shape, and texture, and yielded an average area under the receiver operator characteristics (AUROC or AUC) of 0.83 [0.71, 0.94] for the training cohort. The addition of clinical factors did not improve the predictions, yielding an average AUROC of 0.82 [0.68, 0.94]. The trained predictor was then tested on the independent dataset, which yielded an average AUROC of 0.90 [0.89, 0.91], both with and without clinical factors (Table 3). In this first search, features from different categories were mixed and each of the features was equally likely to have been selected. Subsequently, features were categorized into three broad categories, C1-C3, as described in Methods and the discriminatory analysis was repeated. Table 4 presents the top four-dimensional discriminant features in each of the three categories that were significantly different between the cases and controls. Among features in the size and shape category (C1), the best pair (shortaxis * long diameter, short axis, density, rectangular fit) yielded an average AUROC of 0.80 [0.66, 0.91] in the training cohort and 0.86 [0.84, 0.86] in the test set. Using location-co-occurrence-runlength-histogram category (C2), the best features set (border to lung, relative-3D-voi-airspace, 3D-average-vol, avg. co-occurrence) yielded an average AUROC of 0.85 [0.73, 0.95] in training cohort and 0.88 [0.87, 0.88] in test set. Among features in the texture category (C3), the top feature set (based on wavelets and Laws based textures) yielded an average AUROC of 0.77 in train cohort and 0.86 in the test set. These category-wise prediction analyses help us to evaluate the performance in each of these individual groups. Detailed results are presented in Supplemental Tables S8 and S9.

**Table 3:** Top images features discriminating cancer and benign nodules using image features across categories.

All Categories (129 Reproducible, Non-Redundant Features)					
Features		Training		Testing	
Broad description	Detailed feature	Image	Image + Clinical	Image	Image + Clinical
		E[AUC], CI	E[AUC], CI	E[AUC], CI	AUC
Size, Shape and Texture	(F3: ShortAx; F4: Mn-Hu; F26: EllipticFit; F176: 3D-Laws-127)	0.83 [0.71, 0.94]	0.82, [0.68, 0.94]	0.90 [0.89, 0.91]	0.89 [0.88, 0.9]
Location, Texture	(F19: 10a-3D-Relat-Vol-Airspaces;F21: 10c-3D-Av-Vol-AirSpaces;F22: 10d-3D-SD-Vol-AirSpaces; F201:3D-WaveP2-L2-12)	0.83 [0.70, 0.93]	0.81, [0.67, 0.92]	0.87 [0.86, 0.88]	0.86 [0.85, 0.87]
Location, Size, Texture-Runlength	(F19: 10a-3D-Relat-Vol-Airspaces;F34: Volume-pxl; F37: Thickness-Pxl; F48: AvgGLN)	0.80, [0.65, 0.92]	0.80, [0.68, 0.89]	0.89 [0.89, 0.90]	0.88 [0.869, 0.893
**Baseline**	Longest Diameter	0.76, [0.62, 0.93]		0.85, [0.35, 0.95]	
	Volume	0.78 [0.63, 0.91]		0.87 [0.37, 0.96]	

Statistical estimates were computed using cross validation (hold out) for training and tested, randomized multiple times.

**Table 4:** Top image features discriminating cancer and benign nodules, using feature in each category (size & shape, Texture: co-occurrence, runlength, pixel HU, Texture: wavelets & laws).

Image feature based predictors across different descriptive feature *categories*				
Features	Training		Testing	
	Image	Image + Clinical	Image	Image + Clinical
	E[AUC], CI	E[AUC], CI	E[AUC], CI	E[AUC], CI
**Size & Shape Features** (17 Reproducible, Non-Redundant Features)				
**Radiomics: Size and Shape** (F2: ShortAx-LongDia; F3: ShortAx; F38: Length-Pxl; F32: RectangularFit)	0.80 [0.66, 0.91]	0.78, [0.651, 0.92]	0.86 [0.85, 0.86]	0.85, [0.84, 0.86]
**Size and Shape** (F2: ShortAx-LongDia; F3: ShortAx; F25: Density; F32: RectangularFit)	0.79, [0.65, 0.90]	0.79, [0.60, 0.91]	0.86, [0.85, 0.86]	0.84 [0.83, 0.86]
**Texture: Location, Co-occurrence, RunLength & Pixel HU. Histogram Features** (25 Reproducible, Non-Redundant Features)				
**Location, Cooccurrence, Runlength** (F9: 8b-3D-Bord-to-Lung; F19: 10a-3D-Relat-Vol-Airspaces; F21: 10c-3D-Av-Vol-AirSpaces; F42: AvgCoOc-Homo)	0.85, [0.73, 0.95]	0.84, [0.71, 0.94]	0.88 [0.87, 0.88]	0.87 [0.87, 0.88]
**Location, Cooccurrence, Runlength** (F27: Main-Direction; F185: Hist-SD-L1; F186: Hist-Energy-L1; F187: Hist-Entropy-L1)	0.79, [0.59, 0.93]	0.77, [0.64, 0.93]	0.86 [0.84, 0.87]	0.85 [0.84, 0.86]
**Texture: Laws & Wavelets Features** (86 Reproducible, Non-Redundant Features)				
**Texture by wavelet & Laws** (F72: 3D-Laws-14; F200: 3D-WaveP2-L2-11; F215: 3D-WaveP1-L2-26; F216: 3D-WaveP1-L2-27)	0.761, [0.63, 0.92]	0.75, [0.54, 0.87]	0.79 [0.79, 0.80]	0.774 [0.75, 0.79]
**Texture by wavelet and Laws** (F143: 3D-Laws-94; F201: 3D-WaveP2-L2-12; F214: 3D-WaveP1-L2-25; F216: 3D-WaveP1-L2-27)	0.774, [0.62, 0.9]	0.76, [0.61, 0.9]	0.819 [0.82, 0.83]	0.804 [0.79, 0.82]

Statistical estimates were computed using cross validation (hold out), repeated multiple times.

In the next set of analyses, we focused on applying the image-based predictors in the nodule size range of 4 to1 2 mm and in the large nodule size range of >12 to 30 mm. In the first analysis we used all categories of features restricted to nodules size of 4 to 12 mm, to predict cancer status (see Table 5). The top four-dimensional descriptors were based on size, density and texture (mean HU, elliptic fit, volume, Laws) with an average AUROC of 0.75 in the training, and 0.78 for the test set. At a larger range >12 mm, the top pair of features were co-occurrence and texture (avg cooc-contrast, wavelets-L2-7, wavelets-L2-10, wavelets-L2-25) with an average AUROC of 0.70 and 0.61 in the training and test cohorts, respectively.

**Table 5:** Top images features discriminating cancer and benign nodules across different size ranges, with mixed image categories.

Different nodule size ranges.				
Range: 4 to 12 mm, All Categories (129 Reproducible, Non-Redundant Features)				
Features	Training		Testing	
	Image	Image + Clinical	Image	Image + Clinical
	E[AUC], CI	E[AUC], CI	E[AUC], CI	E[AUC], CI
**Radiomics: Pixel-Hu, Size, Shape & Texture** (F4: Mn-Hu; F26: EllipticFit; F34: Volume-pxl; F172: 3D-Laws-123)	0.75, [0.39, 0.96]	0.74, [0.46, 0.97]	0.78, [0.76, 0.79].	0.75, [0.71, 0.78]
**Radiomics: Pixel-Hu, Shape, Size & Texture** (F4: Mn-Hu; F26: EllipticFit; F34: Volume-pxl; F138: 3D-Laws-89)	0.74, [0.47, 0.95]	0.73, [0.48, 0.96]	0.79 [0.77, 0.81]	0.759, [0.72, 0.79]
**Baseline:** Longest Diameter	0.595, [0.29, 0.87]		0.67, [0.17, 0.86]	
Volume	0.589, [0.33, 0.87]		0.68 [0.51, 0.69]	
**Range: >12 to 30 mm**, **All Categories (129 Reproducible, Non-Redundant Features)**				
**Radiomics: Texture-Cooccurrence, Wavelets** (F44: AvgCooC-Constrast; F196: 3D-WaveP2-L2-7; F199: 3D-WaveP2-L2-10; F214: 3D-WaveP1-L2-25)	0.70, [0.24, 0.96]	0.69, [0.2, 0.95]	0.61, [0.51, 0.65]	0.65, [0.44, 0.65]
**Radiomics: Location, Texture by Cooccurence, Wavelets** (F17: 9f-3D-Min-Dist-COG-to-Border; F44: AvgCooC-Constrast; F204: 3D-WaveP2-L2-15; F214: 3D-WaveP1-L2-25)	0.71, [0.37, 0.97]	0.545, [0.24, 0.86]	0.57, [0.50, 0.6]	0.55, [0.44, 0.65]
**Baseline:** Longest Diameter	0.53 [0.05, 0.90]		0.57, [0.56, 0.57]	
Volume	0.57 [0.07, 0.95]		0.65 [0.34, 0.66]	

Statistical estimates were computed using cross validation (hold out), randomized multiple times.

**Figure 3:**
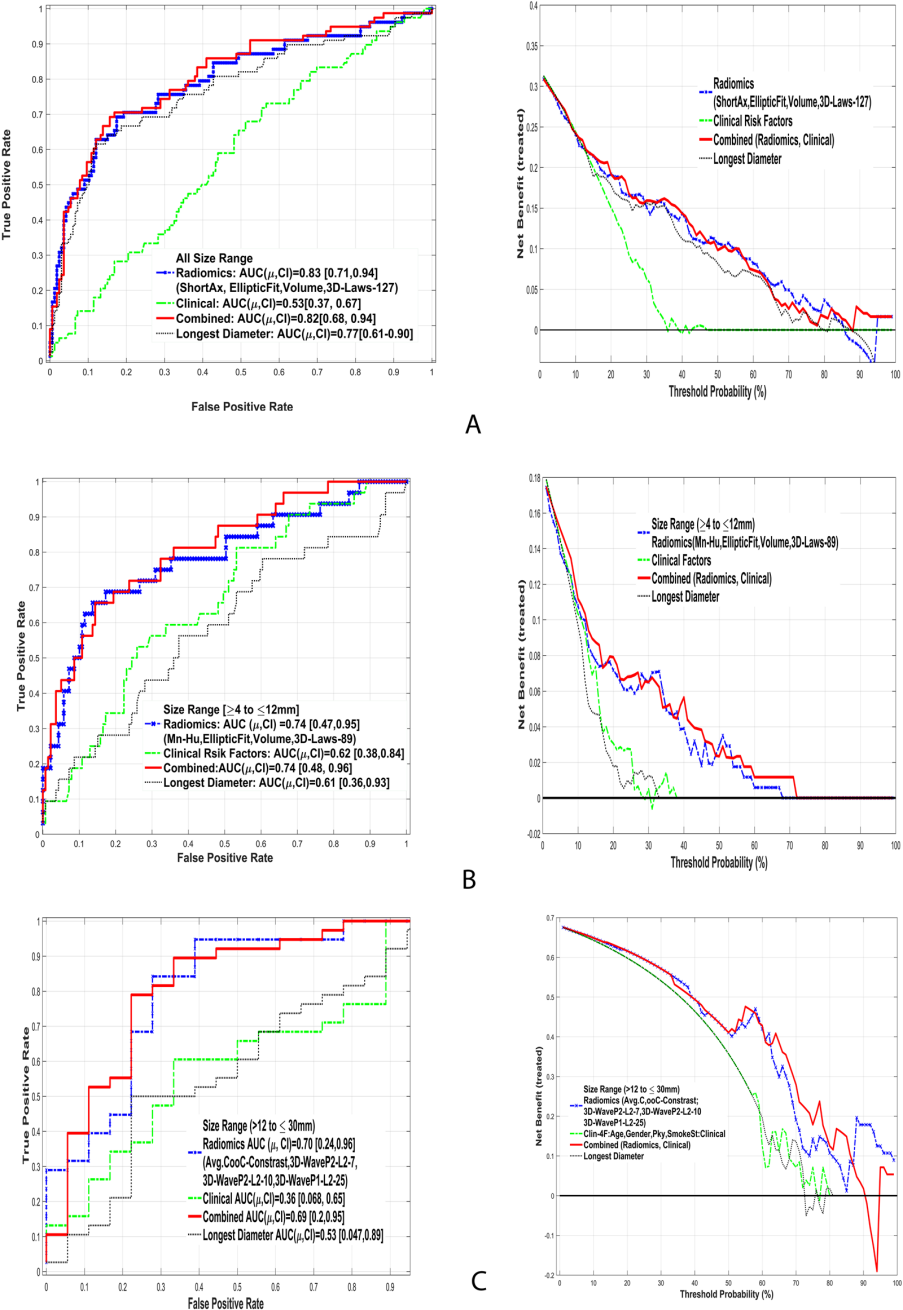
Comparison of area under receiver operator curves (AUC) and clinical net benefit curves using radiomics image markers to predict cancer status in lung nodules with different sizes. (**A**) All size range, (**B**) Indeterminate range (4 to 12 mm), (**C**) larger range (>12 to 30 mm).

The discrimination ability of these image features in two nodule size ranges was subsequently investigated in each of the three feature categories (Table 6). The features in category C2 (border to lung, dist COG, rel-vol_airspace, avg-Run-RP) yielded an average AUROC of 0.83 and 0.76 in the train and test cohorts, respectively. Using the larger nodule size range (>12 to 30 mm), the most discriminant features were also in category C2 (co-occurrence, location, entropy and run-length features), which yielded an average AUROC of 0.67 and 0.68 for the testing and training sets, respectively. Figure 3 shows the AUROC of the top discriminant feature set along with Net clinical benefit analysis for the various size ranges. While the longest diameter based predictor shows lower range of AUROC compared to non-size based radiomic predictors. We evaluated the clinical benefit of the radiomic feature predictors that were found in the classifier analysis and evaluated the effectiveness of these markers under each of the nodule size ranges. Using all size range of nodules, we found the clinical benefit of radiomic prediction is better than clinical factors for a threshold of 20% or greater. These predictors show improvement compared to the nodules longest diameter, in the threshold range of 40% or greater. We then considered the top radiomic predictors in the indeterminate range (≥4 to ≤12 mm). Our analysis found distinct benefit of using a radiomic based predictor compared to conventional size or clinical factors at a threshold of 10% or greater. Among the larger nodule size range (>12 to ≤30 mm) there was a distinct clinical benefit compared to size metric or clinical factors, beginning at a cutoff of 20% or greater.

**Table 6:** Top images features discriminating cancer and benign nodules across different size ranges and features with in each image categories.

Feature categories and nodule size ranges.				
Range: 4 to 12 mm (129 Reproducible, Non-Redundant Features)				
Features	Training		Testing	
	Image	Image + Clinical	Image	Image + Clinical
	E [AUC], CI	E [AUC], CI	E [AUC], CI	E[AUC],CI
**i. Size and Shape Features (C1)**				
**Radiomics: Size & Shape** (F6: Vol-cm; F38: Length-Pxl; F40: Length-by-Width; F41: Border-Leng-Pxl)	0.62, [0.415, 0.811]	0.61, [0.39, 0.82]	0.71 [0.68, 0.72]	0.66, [0.61, 0.69]
**ii. Location, Co-occurrence, Run Length & Pixel HU. Histogram (C2)**				
**Radiomics: Location, Cooccurrence, Runlength** (F9: 8b-3D-Bord-to-Lung; F17: 9f-3D-Min-Dist-COG-to-Border; F19: 10a-3D-Relat-Vol-Airspaces; F55: AvgRP)	0.83, [0.63, 0.97]	0.81, [0.61, 0.97]	0.764 [0.74, 0.78]	0.665 [0.02, 0.67]
**iii. Texture: Laws & Wavelets (C3)**				
**Radiomics: Texture – Laws & Wavelet** (F68: 3D-Laws-10; F201: 3D-WaveP2-L2-12; F214: 3D-WaveP1-L2-25; F216: 3D-WaveP1-L2-27)	0.61, [0.27, 0.89]	0.64, [0.40, 0.86]	0.649 [0.62, 0.66]	0.58, [0.53, 0.625]
**Range: >12 to 30 mm** ** (129 Reproducible, Non-Redundant Features)**				
**i. Size & Shape Features (C1)**				
**Radiomics: Size & Shape** (F37: Thickness-Pxl; F41:Border-Leng-Pxl; F25: Density; F26: EllipticFit)	0.71, [0.19, 0.96]	0.63, [0.25, 0.96]	0.79 [0.72, 0.83]	0.784, [0.70, 0.86]
**ii. Location, Co-occurrence, Run Length & Pixel HU. Histogram (C2)**				
**Radiomics: Location, Cooccurrence, Runlength** (F187: Hist-Entropy-L1; F188:Hist-Kurt-L1; F42: AvgCoOc-Homo; F48: AvgGLN)	0.67, [0.21, 0.96]	0.57, [0.19, 0.88]	0.68, [0.62, 0.715]	0.659, [0.55, 0.76]
**iii. Texture: Laws & Wavelets (C3)**				
**Radiomics: Texture-Laws & Wavelet** (F196: 3D-WaveP2-L2-7; F199: 3D-WaveP2-L2-10; F214: 3D-WaveP1-L2-25; F216: 3D-WaveP1-L2-27)	0.76, [0.42, 0.97]	0.68 [0.28, 0.96]	0.591 [0.55, 0.61]	0.634 [0.57, 0.68]

Statistical estimates were computed using cross validation (hold out), randomized multiple times.

**Figure 4:**
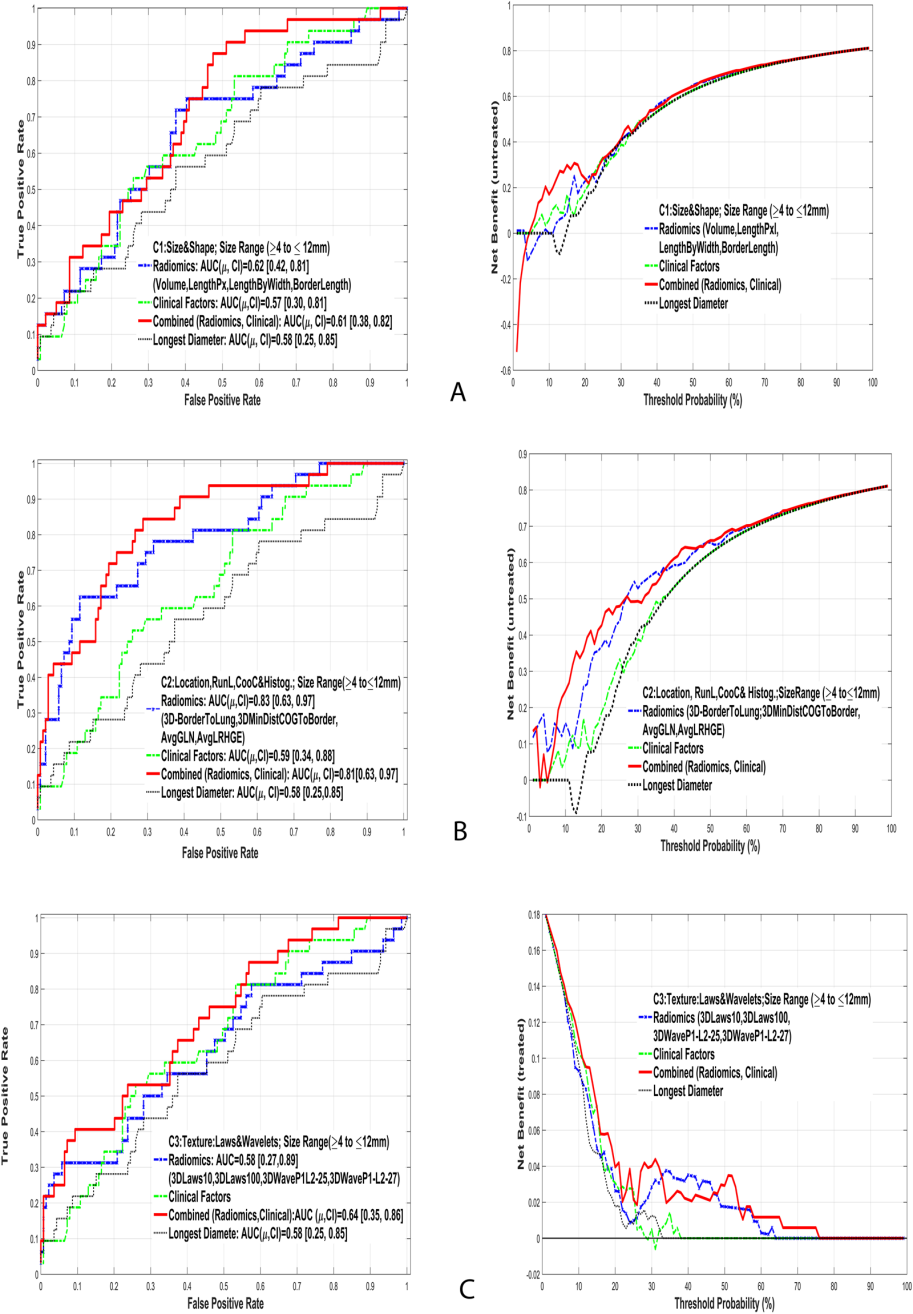
Comparison of area under receiver operator curves and clinical net benefit curves to predict cancer status of lung nodules in the indeterminate range (4 to 12 mm) using different categories of Radiomic features: (**A**) C1: Size & Shape, (**B**) C2: Run length & Co-Occurrence (**C**) C3: Texture (Wavelets & Laws).

Figure 4 shows the top discriminant feature’s performance of the indeterminate size range for different categories. The results for lower and middle range of nodules are also presented. Detailed prediction results are presented in Supplemental Tables S10 and S11. The longest diameter based predictor had an AUROC of around 0.76 [0.62, 0.93] for all range, 0.60 [0.28, 0.87] for intermediate size and 0.53 [0.05, 0.90] for larger size range.

## Discussion

The management of pulmonary nodules is evolving clinical challenge with subjective clinical criteria, especially for IPNs^6,7,17^. Currently guidelines largely consider size and shape characteristics as the predictors of aggressiveness. Using this information along with clinical factors, there have been numerous studies that provide patient level risk assessments^18–20^. Recently, our group used radiological information to provide improved nodule characterization^21^. In the current study, we took a quantitative approach to extract repeatable, non-redundant radiomic features and found combinations of the features in a multivariate setting to be different in cancerous nodules. Our current analysis is an advanced and improved approach in quantifying the variations of the nodule regions at a sub-voxel level to provide malignancy risk assessment. In order to compare with conventional knowledge, we functionally divided the features into categories (C1: size/shape, C2: co-occurrence/run length/location, C3: wavelet/Laws) and investigated the discriminatory ability within and across the groups and demonstrated that adding texture features (non-size based metrics) improved cancer prediction and reduce false positives.

The current clinical management of pulmonary nodules is based on size, volume and doubling time, which are shown to be prognostic^22^. In our analyses the best feature set in the shape and size category had an AUROC of 0.8 (Sensitivity or TPR of 0.44, Specificity or TNR of 0.93, see Table 4 and Supplemental Table S9). In comparison, the best feature set in category 2 (C2: runlength/co occurrence/location) had an AUROC of 0.85 (Sensitivity of 0.54, Specificity of 0.91, see Table 4 and Supplemental Table S9), which showed an improvement of improvement of 6.3% in AUROC. The texture metrics have an equal or better predictive ability compared to conventional size metrics. It also supports our hypothesis that adding textural information added to size and shape metrics will improves predictions. We further restricted the nodules to two size ranges: indeterminate size (4 to ≤12 mm) and intermediate sizes (>12 to 30 mm) and repeated the search for discriminant image features. Due to limited nodule sizes the predictability of the image features showed a drop, compared whole range, with an the AUROC of 0.75 using mixed features (mean-HU, elliptical fit, volume, laws), while for larger nodules (>12 mm) the average AUROC dropped to 0.71 (see Table 5 and Supplemental Table S10). In both these cases, the selected features were dominated by non-texture metrics. Net benefit analyses shows improvement in disease discovery compared to conventional size metrics at a range of cutoffs, allowing the clinical practioner to choose a level based on the clinical context and acceptable false positive rate.

When features are grouped into size range followed by the category types, we found that texture defined by run length/co-occurrence features performed well, even at the indeterminate size range (4 to 12 mm); the average AUROC was 0.83 (see Table 6 and Supplemental Table S11). It is hypothesized that the malignant nodules have patterns of density characteristics (heterogeneity) that are captured by the texture metrics; and that the solidity of the nodule is captured by the mean-HU and finer texture by Laws features. In the above categorizations, textures defined by run length/co-occurrence showed better discrimination compared to finer textures (wavelets and Laws), possibility due to lower levels of inter-tumor variation. Wavelet functions parse the data in multiscale levels by kernel convolution and extracting features in each of these scales describe underlying finer texture. These metrics in medical imaging have shown utility in finding abnormal regions^23^, but their association to radiological measurements are currently unknown. In the context of pulmonary nodules, adding texture metrics to conventional size measure may certainly aid our ability to discriminate both in the indeterminate and full range of pulmonary nodules. The size-based (longest diameter) predictors showed lower performance compared to radiomic predictors in most size-based categories. In our study, patients were randomly split into train and test cohort, the discriminator was formed in the train cohort and applied blindly on the test cohort. Due to limited sample size coupled with diverse patients, it is possible that test AUROC could be better than the train AUROC.

Prior published work has been conducted to compare discrimination metrics to evaluate diagnostic tests; and each presents a set of competing arguments^24,25^. Though there is a lack of strict guidelines for the choices of these approaches, each method has certain limitation on the usage that could be the needed guiding principle. Over-fitting and sample size are two most critical factors that need to be considered before choice of a methods. Many approaches and comparisons of discriminatory methods have been investigated^26,27^. In our study we have taken a conservative approach to choose a linear discriminatory model with an emphasis on the reproducibility of the findings. In addition to rigorous cross validation approach a secondary independent validation was carried out.

Prior studies showed the importance of computer aided diagnosis and texture features in the context of lung nodules^28,29^. Recently, Orizco *et al*.^30^ used 19 texture features comprises of wavelets, co-occurrence and showed accuracy of malignancy prediction to be 82% (sensitivity of 90.1%, specificity 73.9%, AUROC of 0.81) in a cohort of 61 samples for training and 45 for testing. In comparison, we found lower level of sensitivity but relatively higher specificity and AUROC (sensitivity: 39.5%, specificity: 93.6%, AUC 0.761 and test sensitivity of 39.8%, specificity of 93.2% and AUROC of 0.79), for predictor with mixed feature categories (size, texture). While there are some studies that report AUROC of 92.7%^31^, in most of those cases the nodules ground truth was based on radiological assessment, such as in the lung image database consortium (LIDC)^32^. In another reported cross-institutional study^33^, used CT lung nodule cases from The Weill Cornell database (259 cases, 167 cancer, 92 controls) and NLST study (477 cases, 245 cancer, 232 controls), which also compared different types of classifier models. The reported feature model was based on 25 image features using the Support vector machines (SVM) with an AUROC of 0.73 in The Weill Cornell dataset, AUROC of 0.76 in NLST dataset and AUROC of 0.77 in combined (The Weill Cornell and NLST) dataset. The reported image features are mixture of descriptors, based on curvature statistics, margin gradient features, density and morphological features. In comparison, we report different combination of non-texture and texture based features with comparable and superior AUROC performance (AUROC of 0.85).

Implementation differences across research groups and software packages still remain a major issue. Working groups within the NCI Quantitative Imaging Network (QIN), RSNA’s Quantitative Imaging Biomarker Alliance (QIBA) and the Imaging Biomarker Standardization Initiative (IBSI) have undertaken community-wide efforts, some with industry, to establish definitions for radiomic imaging metrics, and sources of variability^34–36^. Scanner parameters, especially reconstruction kernel choices are critical choices that influence the radiomic metrics but hard to control in a clinical setting, we have taken effort to grade the features by its variability and recommend using ones that shows lower within group deviations.

### Combined features

In this study, we used the most common risk factors in lung cancer^37^, including age, sex, pack years smoked and smoking status. In most cases, the clinical models do not improve image based predictors. This could be attributed to the high baseline risk due to heavy smoker population in the NLST data. In our study, the lung cancer case and nodule positive controls were epidemiologically matched followed by randomization to form train and test cohorts to de-couple any unknown patient level effects.

## Materials and Methods

### Study cohort and protocol

After execution of the Data Transfer Agreement (DTA) between the Moffitt Cancer Center and National Cancer Institute, we downloaded LDCT images and patient data from the CDAS (Cancer Data Access System) through the TCIA (The Cancer Imaging Archive) web portal. The study used de-identified patient data, informed consent for retrospective analysis was waived, HIPPA Compliant (Health Insurance Portability and Accountability Act) and approved by the University of South Florida’s Institutional Review Board (IRB), protocol MCC16763.

### Nested study design

We identified 196 screen-detected lung cancers in the LDCT arm of the NLST who had a baseline positive screen that was not determined to be lung cancer and subsequently had a follow-up positive screen that was diagnosed as lung cancer in the first (N = 104) or second (N = 92) follow-up screen. Based on the availability of complete LDCTs or an inability to verify nodule abnormality by the study radiologist (H.W, Y.L), the 196 was reduced to 166 lung cancer cases that were used in this analysis. To create a test set and validation set from the 166 lung cancer cases, we randomly selected 78 subjects as the training set and the remaining 88 were used as the test or validation set. The cohort selection schema and design has been previously described^38^.

Using a 2:1 to nested case-control study design, we identified 392 LDCT screening participants who had three consecutive positive screens that were not diagnosed as lung cancer. These were designated as our nodule–positive (+) controls and as such at respective time of diagnosis represent a participant with a nodule that is not cancer. The nodule + controls were frequency matched to the lung cancer cases, age at randomization (±5 yrs), sex, race/ethnicity, smoking status, pack-years smoked (±5 pack-yrs), and age quit among former smokers (±5 yrs). This design minimizes the influence of confounders between the cases and the nodule + controls, and thus, any quantitative imaging features that differentiate cases and nodule + controls will not likely be attributed to external risk factors. Additionally, the rationale for selecting nodule + as controls was that these individuals had the same screening clinical characteristics as those who developed lung cancer. Based on the availability of complete LDCT images and consensus to identify the nodules across time points, the original set of 392 nodule + controls were reduced to 313. To create a test set and validation set from the 313 nodule + controls, we randomly selected 166 subjects as the training set and the remaining 147 were used as the test or validation set. The clinical characteristics of the cohorts and the reconstruction kernel choices for LDCT are presented in Tables 1 and 2 and detailed scanner parameters for the cohorts are presented in Supplemental Table S1. In our analysis, we added a randomization step for selecting test and train cohort to avoid any patient level influence on the image based biomarker analysis.

### Nodule identification and quantitative imaging features

The study radiologists with over 6 year clinical experience (HW and YL) verified NLST radiologist provided location (slice number and anatomical location) of nodules on the LDCT images. The control subjects determined as non-cancerous by NLST, could have had either non-growing nodules or were pathologically verified as benign. The research radiologists used the baseline scan to identify consensus abnormalities for nodule-positive controls. When multiple nodules were present for participant, we used the largest nodule, measured by longest diameter to choose one nodule per patient, our prior study describes the radiological aspects of the cohort^39^.

The locations of the nodules for the lung cancer cases and nodule(+) controls were verified by the radiologists and were used to seed a semi-automated single click ensemble segmentation (SCES) algorithm^40^ to segment and render the nodule. These nodule boundaries were verified by the study radiologist. If the nodule was attached to pleura or mediastinal wall, the semi-automated segmentation often resulted in improper boundary and was subsequently altered based on the radiologists’ opinion. In prior work, we have shown ensemble of various segmentation regions is superior to achieve most probable regions of interest in lung lesions^41^. These regions may contain all observable regional variations such as solid, part-solid ground-glass opacities (GGOs), and mixed cases. The common segmentation issues encountered and consensus solutions that were followed in the study are enumerated in Supplemental Table S2. Current tumor delineation challenges and the state of research have recently been reviewed^42^. From the segmented regions, 219 quantitative features were extracted with descriptors from three broad categories. Category 1 (C1) features described the tumor region’s shape (e.g. ellipsoid, round) and size (e.g. volume, diameter). Both of these feature categories are computer derived and hence, structured. In current radiology practice, size based features (e.g. longest diameter) are captured as structured data, whereas other features are not (e.g. spiculated, lobulated). These features also have direct relationships to the tumor dimensions (size and shape) that are currently used in clinical practice. Category 2 (C2) features described regional and spatial textures, such as run lengths, co-occurrence matrices, pixel histograms, and location. These features could be qualitatively related to conventional radiological description such as non-homogeneous appearing nodules. Category 3 (C3) features quantified more subtle textural variations described by wavelets and Laws based features^43^. These features cannot be visualized by naked eye, but they do provide a finer ensemble measurement for the intra-tumor heterogeneity. These features are computed in the transformed domain by kernel functions. Supplemental Tables S3 and S4 list the computer-generated feature categories and provide background descriptions.

### Repeatable image features

Image features was filtered based on their repeatability, calculated from a test-retest data from The Reference Image Database to Evaluate Therapy Response (RIDER)^44^ dataset. In a prior study^23,43^, we have shown that a subset of image features are repeatable in test-retest CT images obtained twice on the same patient with in a short break (referred to as “coffee break” experiment), where the variability in patient and scanning procedures was minimized. We used concordance correlation coefficient (CCC) between test and re-test to filter features to quantify repeatability, which measures the variability from the absolute diagonal line. Other metrics such as the inter-class concordance coefficient (ICC) and repeatability coefficient (RC) can be utilized to quantify repeatability. The most common form of ICC measures the difference between two measurements and the average difference over time. While the repeatability coefficient (RC) measure the bound for the population, which is related to 95% limits of agreement on the true difference between measurements. In the present study, we also interrogated features from known benign cases across all three time points to assess repeatability. We set the CCC ≥ 0.7 as limit on the repeatability, which resulted in 141 features from the initial set of 219. Redundancy between features was quantified by computing the coefficient of determination (*R*^2^). The dependent features within a given set with *R*^2^ ≥ 0.95 were combined into groups and replaced with one representative feature from that group that had highest variability (dynamic range). The filtering steps resulted in 129 features, which were considered to be repeatable and non-redundant in the data set.

### CT scanner reconstruction kernels and influence on image features

In a recent study, the RIDER data set was reconstructed at different imaging settings, varying slice thicknesses and reconstruction algorithms. Reproducibility of the image features was assessed across the scanner variables and the test-retest scans across the population. The authors found most image features were repeatable across different slice thickness. But the feature variability between different reconstruction kernels seem to be very high^45^.

CT scanner manufacturers use proprietary kernel functions to reconstruct images from the sinogram data generated from multiple detectors within the scanner gantry. The optimization criteria for the reconstruction algorithm were the generation of visually useful images for expert radiologists. Hence, there is a need to study the effects of image reconstruction and slice thickness on the downstream analyses. Recently there have been some published studies on the influence of scanner parameters on quantitative image features^46–49^. The Radiological Society of North America (RSNA)’s community effort led through the Quantitative Imaging Biomarker Alliance (QIBA) to understand the scanner kernel functions, make comparisons within and between CT manufacturers. These differences may likely be tolerated by clinical radiologists as their interpretations are relatively immune to the scanner parameter differences. In this study, we investigated the change in reconstruction parameter and its effects on the prediction results. Supplemental Fig. SF2 shows the work flow designed for our current assessment of the impact of reconstruction kernels on extracted features. There are scanner parameters, such as scanner technology/types, pitch, tube voltage, tube currents, field of view that may have some influence on the image descriptors, but are not considered here, as they have recently been addressed in a related study^50^. To investigate the effects of reconstruction kernel, we used an ad hoc approach to determine how this affected the predictive value using one reconstruction to train and another to test. We assembled a cohort of 148 patient scans from the NLST, that were each reconstructed with two different convolution kernels, described in Supplemental Table S5. We evaluated the prediction variability for these training/testing paired kernel cohorts. We tested on these pairs of the manufacturer kernels: Siemens 30 F/2 mm vs. Siemens 50 F/2 mm; and GE Standard/2.5 mm vs. GE Bone/2.5 mm. Supplemental Tables S6 shows the difference in prediction AUROC values due to difference in two reconstructions of matched image pairs, along with feature descriptors.

It is interesting to note about 64.7% of size based features showed less than 5% variability in AUROC, and 76.5% of them showed less than 10% variability comparing Siemens reconstruction algorithms. On GE scanners, there were 47.1% features that showed less than 5% variability and 82.4% features with less than 10% variability. Similar proportions held for features defined by tumor location, run length, co-occurrences and pixel histogram (46.2% features on Siemens and 42.3% features on GE had less than 5% variability). The texture defined by wavelets and Laws based texture features showed minimal change in AUROC, this is likely due to the fact that features with-in group (cancer or normal) variability was so high that the discriminatory ability (cancer to normal) of these features was lost. The differences in prediction performances (AUROC) are reported in Supplemental Table S7. We found that there were sub set of features that can withstand scanner parameter variability.

Due to limited choices of consistent scanner types, we made a qualitative assessment of different scanners following the recommendations by the study radiologists and used a comparable reconstruction kernels mapping across manufacturers. Our choice was to find a scan from the same manufacturer scanner setting following the choices in the table (‘followed choice’, see Table 2).

### Statistical analysis

We used optimal linear classifier model to discriminate benign from malignant nodules in a cross validating setting^51^. A hold out type error estimation procedure was followed with 80% of the samples used for training and remaining for testing, the cross validation process was randomly repeated 200 times. The best four-feature sets were identified on the repeatable and non-redundant feature set, sorted based on Youden’s J-Index. This allows selection of image features that are optimized for sensitivity, specificity. In multivariate settings, using features that complement each other is expected to improve the discriminatory performance. Numerous suboptimal methods have been proposed to find best set of features for a discriminatory problem, some of the methods have been reviewed in the context of quantitative imaging^52^. Most often the discriminators are referred to as a set of n-features or as n-dimension feature pair or features set. In our study we limited our search to find not more than 4 features in multivariate setting, so as to avoid model over fitting. The training error drops as number of features in the model increases, while the J-index tends to show upper trend as we approach four features (see Supplemental Table S8a). The feature space was exhaustively searched taking all possible combinations at respective dimension. This will be close to optimal search for the given set of samples, for 129 features the search space involves testing about 11 million sets of 4-dimensional features, optimized to run on a high performance computing environment. There are several dimensionality reduction method proposed in the literate, some of those claim to be close to optimal^53,54^. In this first search, features from different categories were mixed and they were equally likely to be selected. The reported features are the ones that performed best in the training set and validate in the independent test set. At every feature set, different types of small sample error estimates were computed (hold-out, leave one out, 10-fold, bootstrap cross-validation)^55^.

The top discriminating features set were tested on the independent test data that was not part of training set. In a screening setting, the tradeoffs between sensitivity and specificity can be assessed with a decision curve analysis. Typically, classifier optimizing is based on one of the metrics (like accuracy), which allows bias in either false positive or negatives. The ability to find optimal features that perform on independent test samples relies on optimizing both the false positives and false negative rates. Doing this using an AUROC approach allows finding discriminant feature sets and provides the practitioners to identify optimal cutoff points. Statistical methods were implemented as custom routines written in C/C++ and Matlab®. Our methodology and inference complies with the recommendation by the TRIPOD^56^ to the highest level of validation in an independent dataset.

In recent advancement there has been an emergence of prediction models, need for clinical translation has seen development of net-benefit or cost of decision approaches^57^. In our analysis, we computed the net-benefit of treatment in finding the true disease with range of cutoffs, for the top performing radiomic predictor. These models were contrasted with the most common clinical feature, longest diameter of the nodule.

### Study workflow

In this study we investigated the performance of quantitative image features as a decision support tool in the context of lung screening. Traditionally, size based features are routinely used as patient risk assessment. As new texture based quantitative features are being proposed, it becomes imperative to compare the discriminatory performance within and across sub-categories of features and nodule sizes. Following the motivation, we grouped the features into categories that are contextually similar and we compared the performance within and across categories. In the first section of reported results, we use all 129 of the repeatable features to find best discriminant pair. Subsequent analyses used the top discriminant features, which were searched within all three of the feature categories. We then repeated the analysis across different nodule size ranges in two groups: R1 (4–12 mm) and R2 (>12 mm) measured as longest diameter to minimize size bias in the results. We also report performance of clinically relevant univariate predictor formed using longest diameter and volume of the nodule for different size range.

**Figure 5:**
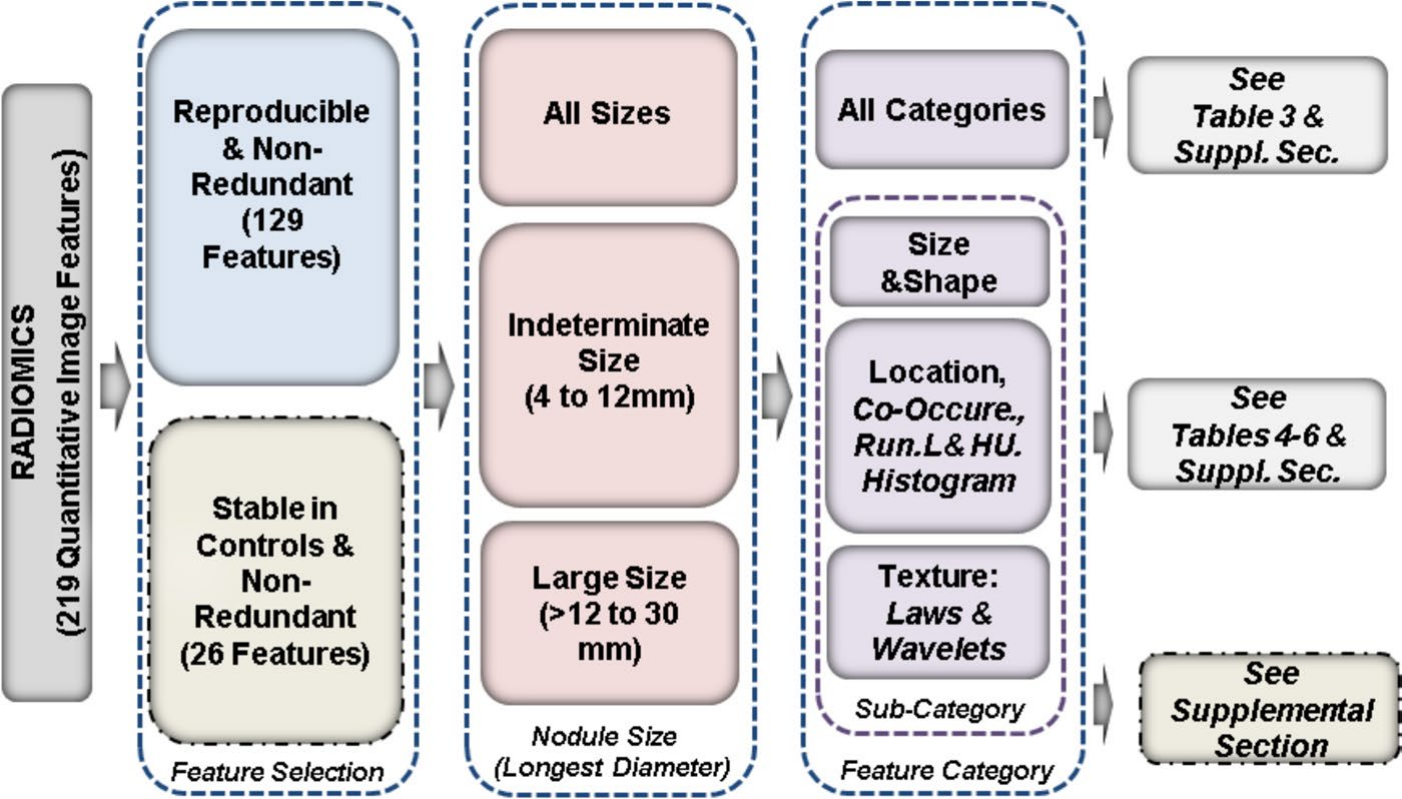
Analysis workflow followed in the study. The extracted features are filtered for reproducibility and redundancy. The data is partitioned into three different sizes based on its longest diameter and further stratified based on feature categories.

The NCCN lung cancer screening guidelines^6^ and Lung-RADS^7^ are commonly utilized guidelines in the U.S for the management of pulmonary nodules detected in the lung cancer screening setting. Both of these recommendations suggest size range for indeterminate pulmonary nodule between 4 to 8 mm with a solid nodule density. If the nodule is part-solid, the longest diameter is suggested to go up to 10 mm. In our study, we included both solid and part-solid components in the region of interest. Because boundary decisions for part-solid nodules are commonly subjective with inter-observer segmentation ranges from 20 to 40%, we relaxed the size range for the indeterminate size to go up to 12 mm. This also allows us to obtain considerable samples for train/test cohort. In our cohort there were 171 patients with nodules between 4 to 12 mm (in training). Figure 5 shows the analysis workflow followed in the study.

## Conclusion

In this study we show quantitative image features can discriminate benign from malignant pulmonary nodules. These descriptors show better prediction of malignancy compared to lung cancer related clinical risk factors by themselves in most settings. The non-size (texture) based quantitative features show better discrimination in predicting cancer status. In addition, adding size based features to texture metrics help improve sensitivity/specificity (or reducing false positives) in predicting malignancy status of the pulmonary nodules. The clinical benefit analysis demonstrates a clear improvement in decisions using radiomic feature based predictors.

## Supplementary information

Supplemental document
