# Supplementary material for: Quantitative Imaging features Improve Discrimination of Malignancy in Pulmonary nodules
Source: Sci Rep. 2019 Jun 12;9:8528. doi: 10.1038/s41598-019-44562-z (PMC6561979; doi:10.1038/s41598-019-44562-z)
Supplement: Supplementary file 1 — Supplemental document [file 41598_2019_44562_MOESM1_ESM.pdf]

SUPPLEMENTAL SECTION
FOR

Quantitative Imaging features Improve Discrimination of Malignancy in Pulmonary nodules

Yoganand Balagurunathan[1], Matthew B. Schabath[2], Hua Wang[1,3], Ying Liu[1,3], Robert J. Gillies[1].

[1]Department of Cancer Imaging and Metabolism and [2]Department of Cancer Epidemiology, H. Lee Moffitt Cancer Center and Research Institute, Tampa, FL.
[3]Department of Radiology, Tianjin Medical University and Cancer Hospital, China.

Corresponding Author
Dr. Yoga Balagurunathan,
Cancer Imaging and Metabolism,
Moffitt Cancer Center,
12901 USF Magnolia Avenue, SRB-4 Imaging,
Tampa, FL 33612.
Email: {yogab, Robert.gillies}@moffitt.org

Keywords: Radiomics on Lung Nodules, IPN, quantitative imaging, CAD methods in pulmonary nodules

Key: Abbreviations used in the study:

A.Classifier Results Section:

Error: Classifier error (1- Accuracy)
Sensitivity(or Recall): True Positive Rate (TPR) =>(True Positive/True Positive + False Negative)
Specificity: True Negative Rate (TNR) => (True Negative/True Negative + False Positive)
PPV (or Precision): Positive predictive value: => (True Positive/True Positive + False positive)
NPV: Negative Predictive value => (True negative/True negative + False negative)
AUC: Area under the Receiver Operator Curve
CI: 95% Confidence Limits (on AUC).

B. Nodule Size

Mixed/All: All size range
Range 1 (R1): Nodule size: ≥ 4 to ≤ 12mm (indeterminate range)
Range 2 (R2): Nodule size: >12 to ≤ 30mm (intermediate range)

C. Feature Categories

Mixed/All: Mixed categories (129 re-producible & non redundant features)
C1: Size & Shape Features (17 features)
C2: Location, Co-occurrence, Runlength, Histogram (26 Features)
C3: Texture: Wavelets & Laws (86 features)
Stable in Controls: Features stable in Nodule positive (Nodule +) controls (26 features)

Definiens Suite (1): Commercial imaging suite used to read and segment CT thorax images. We extract quantitative features using custom routines.

Table S1. Details on CT scanner parameters for case-control cohorts used in the study.

a) Patient distribution for train and test cohort

a. Patient Sample Distribution (Scanner Parameters)							
	**Training**				**Testing**		
	Cancer	Normal	Total		Cancer	Normal	Total
**Samples**	78	166	244		88	147	235
**NLST Trial: Collection Center**							
**LSS** (Lung screening Study)	48	126	174		59	105	164
**ACRIN** (American College of Radiology Imaging Network)	30	40	70		29	42	71
**Scanner Types**					**Scanner Types**		
GE	41	86	127		50	70	120
Siemens	31	59	90		31	52	83
Toshiba	3	7	10		5	7	12
Philips	3	14	17		2	18	20
**Slice Thickness (mm)**					**Slice Thickness (mm)**		
1	1	1	2		0	0	0
1.25	6	5	11		5	6	11
2	31	64	95		36	56	92
2.5	35	80	115		45	72	117
3	2	2	4		0	2	2
3.2	3	14	17		2	13	15
**Reconstruction Kernel**					**Reconstruction Kernel**		
Bone (GE)	1	7	8		0	8	8
Standard (GE)	40	48	88		50	69	119
Lung (GE)	0	1	1		0	1	1
B30f (Siemens)	31	58	89		30	55	85
B50f (Siemens)	0	1	1		1	1	2
FC01(Toshiba)	0	1	1		1	1	2
FC02(Toshiba)	0	1	1		0	0	0
FC10(Toshiba)	0	2	2		3	2	5
FC30(Toshiba)	2	1	3		0	1	1
FC51(Toshiba)	1	2	3		1	3	4
A (Philips)	1	0	1		0	1	1
C (Philips)	0	13	13		2	12	14
D (Philips)	2	1	3		0	0	0

b) Patient distribution based on case and control.

a. Case Control (Scanner & Reconstruction kernels)							
	**Cancer Cases**				**Normal Cases**		
	Train	Test	Total		Train	Test	Total
**Samples**	78	88	165		166	147	313
**Collection Center**							
**LSS** (Lung screening Study)	48	126	174		59	105	164
**ACRIN**(American College of Radiology Imaging Network)	30	40	70		29	42	71
**Scanner**					**Scanner**		
GE	41	50	91		86	70	156
Siemens	31	31	61		59	52	111
Toshiba	3	5	8		7	7	14
Philips	3	2	5		14	18	32
**Slice Thickness (mm)**					**Slice Thickness (mm)**		
1	1	0	1		1	0	1
1.25	6	5	11		5	6	11
2	31	36	66		64	56	120
2.5	35	45	80		80	72	152
3	2	0	2		2	2	4
3.2	3	2	5		14	13	27
**Reconstruction Kernel**					**Reconstruction Kernel**		
Bone (GE)	1	0	1		7	8	15
Standard (GE)	40	50	90		48	69	117
Lung (GE)	0	0	0		1	1	2
B30f (Siemens)	31	30	60		58	55	113
B50f (Siemens)	0	1	1		1	1	2
FC01(Toshiba)	0	1	1		1	1	2
FC02(Toshiba)	0	0	0		1	0	1
FC10(Toshiba)	0	3	3		2	2	4
FC30(Toshiba)	2	0	2		1	1	2
FC51(Toshiba)	1	1	2		2	3	5
A	1	0	1		0	1	1
C	0	2	2		13	12	25
D	2	0	2		1	0	1

b. **Case Control (Tube Voltage & Current)**							
**Cancer Cases**					**Normal Cases**		
	Train	Test	Total		Train	Test	Total
**Tube Voltage (KVP)**					**Tube Voltage (KVP)**		
120	75	152	227		78	143	221
140	3	14	17		10	11	21
**Tube Current (ma)**					**Tube Current (ma)**		
40	0	1	1		0	1	1
50	10	13	23		14	9	23
60	4	13	17		5	16	21
67	1	0	1		0	0	0
70	1	4	5		4	5	9
75	3	15	18		3	19	22
80	18	22	40		17	19	36
90	12	16	28		9	21	30
93	1	9	10		2	9	11
100	6	4	10		2	3	5
105	0	0	0		4	1	5
102	1	1	2		0	0	0
108	1	0	1		0	1	1
117	0	0	0		0	2	2
120	8	10	18		9	19	28
140	2	0	2		2	1	3
150	5	3	8		3	1	4
160	5	23	28		13	26	39
187	0	1	1		0	0	0
417	0	0	0		0	1	1

Supplemental Table S2. Common Segmentation Complications

Complication	Description
**Nodule attached to lung wall**	Often the lung field preprocessing will identify any nodules attached to the lung wall as body tissue
**Semisolid Nodules**	When selecting a seed point in tissue composed of varied intensities (GGO + Solid) Definiens software suite (1) will sometimes select the entire area of interest while other times only selecting the solids, leaving out the GGO
**GGO**	Ground glass opacity nodules will segment incorrectly depending on the intensity of the surrounding tissue. In some cases the “click and grow” will yield no results. (Error Message: Area too Dark)
**Inflammation / Scar tissue**	When selecting a GGO the “click and grow” will lock onto nearby inflammation with brighter intensity. When selecting a solid nodule the “click and grow” will lock onto nearby scar tissue with brighter intensity.
**Contains air**	If a larger nodule has a dark center the “click and grow” routine will try to segment it out but the result is usually quite incorrect; removing tissue that should be included.
**Spiculation**	Generally the more highly spiculated nodules will present more difficulty, and will have a greater chance of selecting nearby bronchi
**Between two lobes**	A nodule can sometimes attach itself to a fissure line between two lobes, allowing it to “move” more than nodules normally do when scrolling through a series.
**Bronchial Tree**	If the bronchial tree is anywhere near the tumor nodule it is almost always included when growing the ROI
**“Independent” nodules too close**	Two independent nodules at T0 can intersect later at T2 and be mostly impossible to differentiate.
**Bone/ Calcification**	If nodule contains a calcification or is near a bone and that tissue is included, the features can be highly skewed by even a pixel or two.

Supplemental Table S3. Feature categories with description.

**Category**		**Description**	**Number of Descriptors**		
	Sub category		Subtotal	Subtotal	Test-retest (CCC≥0.7 & $R^2 \geq 0.95$)
**C1. Tumor Size & Shape**	a: Tumor Size	Size, volume descriptors	13	25	17
	b: Tumor Shape	Roundness/circularity descriptors	12		
**C2:Tumor Location, Co-occurrence, Run L and Histogram.**	a: Tumor Location	Relative to pleural wall, border flags	14	39	26
	b: Pixel Intensity Histogram	Statistics on the Intensity or Attenuation values (in HU)	8		
	c: Grayscale: Runlength & CoOccurrence	Run length and Co-occurrence patterns	17		
**C3: Texture: Laws & Wavelets**	a: Texture: Laws features	Laws Kernel (energy)	125	155	86
	b: Texture: Wavelets	Wavelet kernels (entropy and energy)	30		
**Total**			**219**		**129**

Supplemental S.4 Quantitative Image features used to describe the lung tumors*.

Index	Feature Index	Description of the Features	Feature Category
1	F1	LongDia	C1.Tumor Size & Shape (Size Information)
2	F2	ShortAx-LongDia	
3	F3	ShortAx	
4	F6	Vol-cm	
5	F33	Area-Pxl	
6	F34	Volume-pxl	
7	F35	Num-Pxl	
8	F36	Width-Pxl	
9	F37	Thickness-Pxl	
10	F38	Length-Pxl	
11	F39	Length-by-Thick	
12	F40	Length-by-Width	
13	F41	Border-Leng-Pxl	
14	F7	5a-3D-MacSpic	C1.Tumor Size & Shape (Shape Information)
15	F13	9b-3D-Circularity	
16	F14	9c-3D-Compact	
17	F23	Asymmetry	
18	F24	Compactness	
19	F25	Density	
20	F26	EllipticFit	
21	F28	Rad-Largest-Enclosed-Ellipse	
22	F29	Rad-Smallest-Enclosed-Ellipse	
23	F30	Shape-Index	
24	F31	Roundness	
25	F32	RectangularFit	
26	F8	8a-3D-Attch-Pleural	C2. Location, RunL, CoOcc & Histogram (Location Information)
27	F9	8b-3D-Bord-to-Lung	
28	F10	8c-3D-Bord-to-Pleural	
29	F11	8d-3D-Rat-Free-to-Attach	
30	F12	9a-3D-FractionalAnisotropy	
31	F15	9d-3D-AV-Dist-COG-to-Border	
32	F16	9e-3D-SD-Dist-COG-to-Border	
33	F17	9f-3D-Min-Dist-COG-to-Border	
34	F18	9g-3D-Max-Dist-COG-to-Border	
35	F19	10a-3D-Relat-Vol-Airspaces	
36	F20	10b-3D-Num-AirSpaces	
37	F21	10c-3D-Av-Vol-AirSpaces	
38	F22	10d-3D-SD-Vol-AirSpaces	
39	F27	Main-Direction	
40	F4	Mn-Hu	C2. Location, RunL, CoOcc & Histogram (Pixel Intensity Histogram)
41	F5	Std-Hu	
42	F184	Hist-Mean-L1	
43	F185	Hist-SD-L1	

44	F186	Hist-Energy-L1	
45	F187	Hist-Entropy-L1	
46	F188	Hist-Kurt-L1	
47	F189	Hist-Skew-L1	
48	F42	AvgCoOc-Homo	C2. Location, RunL, CoOcc & Histogram (Runlength and Cooccurrence)
49	F43	AvgCooc-Mp	
50	F44	AvgCooC-Constrast	
51	F45	AvgCooC-Energy	
52	F46	AvgCooC-Entropy	
53	F47	AvgCoOc-Mean	
54	F48	AvgGLN	
55	F49	AvgHGRE	
56	F50	AvgLGRE	
57	F51	AvgLRE	
58	F52	AvgLRHGE	
59	F53	AvgLRLGE	
60	F54	AvgRLN	
61	F55	AvgRP	
62	F56	AvgSRE	
63	F57	AvgSRHGE	
64	F58	AvgSRLGE	
59	F59	3D-Laws-1(E5 E5 E5 Layer 1)	C3.Texture: Laws & Wavelet Feature (with different convolution filters)
60	F60	3D-Laws-2(E5 E5 L5 Layer 1)	
61	F61	3D-Laws-3(E5 E5 R5 Layer 1)	
62	F62	3D-Laws-4(E5 E5 S5 Layer 1)	
63	F63	3D-Laws-5(E5 E5 W5 Layer 1)	
64	F64	3D-Laws-6(E5 L5 E5 Layer 1)	
65	F65	3D-Laws-7(E5 L5 L5 Layer 1)	
66	F66	3D-Laws-8(E5 L5 R5 Layer 1)	
67	F67	3D-Laws-9(E5 L5 S5 Layer 1)	
68	F68	3D-Laws-10(E5 L5 W5 Layer 1)	
69	F69	3D-Laws-11(E5 R5 E5 Layer 1)	
70	F70	3D-Laws-12(E5 R5 L5 Layer 1)	
71	F71	3D-Laws-13(E5 R5 R5 Layer 1)	
72	F72	3D-Laws-14(E5 R5 S5 Layer 1)	
73	F73	3D-Laws-15(E5 R5 W5 Layer 1)	
74	F74	3D-Laws-16(E5 S5 E5 Layer 1)	
75	F75	3D-Laws-17(E5 S5 L5 Layer 1)	
76	F76	3D-Laws-18(E5 S5 R5 Layer 1)	
77	F77	3D-Laws-19(E5 S5 S5 Layer 1)	
78	F78	3D-Laws-20(E5 S5 W5 Layer 1)	
79	F79	3D-Laws-21(E5 W5 E5 Layer 1)	
80	F80	3D-Laws-22(E5 W5 L5 Layer 1)	
81	F81	3D-Laws-23(E5 W5 R5 Layer 1)	
82	F82	3D-Laws-24(E5 W5 S5 Layer 1)	
83	F83	3D-Laws-25(E5 W5 W5 Layer 1)	
84	F84	3D-Laws-26(L5 E5 E5 Layer 1)	
85	F85	3D-Laws-27(L5 E5 L5 Layer 1)	
86	F86	3D-Laws-28(L5 E5 R5 Layer 1)	
87	F87	3D-Laws-29(L5 E5 S5 Layer 1)	

88	F88	3D-Laws-30(L5 E5 W5 Layer 1)	
89	F89	3D-Laws-31(L5 L5 E5 Layer 1)	
90	F90	3D-Laws-32(L5 L5 L5 Layer 1)	
91	F91	3D-Laws-33(L5 L5 R5 Layer 1)	
92	F92	3D-Laws-34(L5 L5 S5 Layer 1)	
93	F93	3D-Laws-35(L5 L5 W5 Layer 1)	
94	F94	3D-Laws-36(L5 R5 E5 Layer 1)	
95	F95	3D-Laws-37(L5 R5 L5 Layer 1)	
96	F96	3D-Laws-38(L5 R5 R5 Layer 1)	
97	F97	3D-Laws-39(L5 R5 S5 Layer 1)	
98	F98	3D-Laws-40(L5 R5 W5 Layer 1)	
99	F99	3D-Laws-41(L5 S5 E5 Layer 1)	
100	F100	3D-Laws-42(L5 S5 L5 Layer 1)	
101	F101	3D-Laws-43(L5 S5 R5 Layer 1)	
102	F102	3D-Laws-44(L5 S5 S5 Layer 1)	
103	F103	3D-Laws-45(L5 S5 W5 Layer 1)	
104	F104	3D-Laws-46(L5 W5 E5 Layer 1)	
105	F105	3D-Laws-47(L5 W5 L5 Layer 1)	
106	F106	3D-Laws-48(L5 W5 R5 Layer 1)	
107	F107	3D-Laws-49(L5 W5 S5 Layer 1)	
108	F108	3D-Laws-50(L5 W5 W5 Layer 1)	
109	F109	3D-Laws-51(R5 E5 E5 Layer 1)	
110	F110	3D-Laws-52(R5 E5 L5 Layer 1)	
111	F111	3D-Laws-53(R5 E5 R5 Layer 1)	
112	F112	3D-Laws-54(R5 E5 S5 Layer 1)	
113	F113	3D-Laws-55(R5 E5 W5 Layer 1)	
114	F114	3D-Laws-56(R5 L5 E5 Layer 1)	
115	F115	3D-Laws-57(R5 L5 L5 Layer 1)	
116	F116	3D-Laws-58(R5 L5 R5 Layer 1)	
117	F117	3D-Laws-59(R5 L5 S5 Layer 1)	
118	F118	3D-Laws-60(R5 L5 W5 Layer 1)	
119	F119	3D-Laws-70(R5 R5 E5 Layer 1)	
120	F120	3D-Laws-71(R5 R5 L5 Layer 1)	
121	F121	3D-Laws-72(R5 R5 R5 Layer 1)	
122	F122	3D-Laws-73(R5 R5 S5 Layer 1)	
123	F123	3D-Laws-74(R5 R5 W5 Layer 1)	
124	F124	3D-Laws-75(R5 S5 E5 Layer 1)	
125	F125	3D-Laws-76(R5 S5 L5 Layer 1)	
126	F126	3D-Laws-77(R5 S5 R5 Layer 1)	
127	F127	3D-Laws-78(R5 S5 S5 Layer 1)	
128	F128	3D-Laws-79(R5 S5 W5 Layer 1)	
129	F129	3D-Laws-80(R5 W5 E5 Layer 1)	
130	F130	3D-Laws-81(R5 W5 L5 Layer 1)	
131	F131	3D-Laws-82(R5 W5 R5 Layer 1)	
132	F132	3D-Laws-83(R5 W5 S5 Layer 1)	
133	F133	3D-Laws-84(R5 W5 W5 Layer 1)	
134	F134	3D-Laws-85(S5 E5 E5 Layer 1)	
135	F135	3D-Laws-86(S5 E5 L5 Layer 1)	
136	F136	3D-Laws-87(S5 E5 R5 Layer 1)	
137	F137	3D-Laws-88(S5 E5 S5 Layer 1)	
138	F138	3D-Laws-89(S5 E5 W5 Layer 1)	
139	F139	3D-Laws-90(S5 L5 E5 Layer 1)	
140	F140	3D-Laws-91(S5 L5 L5 Layer 1)	

141	F141	3D-Laws-92(S5 L5 R5 Layer 1)	
142	F142	3D-Laws-93(S5 L5 S5 Layer 1)	
143	F143	3D-Laws-94(S5 L5 W5 Layer 1)	
144	F144	3D-Laws-95(S5 R5 E5 Layer 1)	
145	F145	3D-Laws-96(S5 R5 L5 Layer 1)	
146	F146	3D-Laws-97(S5 R5 R5 Layer 1)	
147	F147	3D-Laws-98(S5 R5 S5 Layer 1)	
148	F148	3D-Laws-99(S5 R5 W5 Layer 1)	
149	F149	3D-Laws-100(S5 S5 E5 Layer 1)	
150	F150	3D-Laws-101(S5 S5 L5 Layer 1)	
151	F151	3D-Laws-102(S5 S5 R5 Layer 1)	
152	F152	3D-Laws-103(S5 S5 S5 Layer 1)	
153	F153	3D-Laws-104(S5 S5 W5 Layer 1)	
154	F154	3D-Laws-105(S5 W5 E5 Layer 1)	
155	F155	3D-Laws-106(S5 W5 L5 Layer 1)	
156	F156	3D-Laws-107(S5 W5 R5 Layer 1)	
157	F157	3D-Laws-108(S5 W5 S5 Layer 1)	
158	F158	3D-Laws-109(S5 W5 W5 Layer 1)	
159	F159	3D-Laws-110(W5 E5 E5 Layer 1)	
160	F160	3D-Laws-111(W5 E5 L5 Layer 1)	
161	F161	3D-Laws-112(W5 E5 R5 Layer 1)	
162	F162	3D-Laws-113(W5 E5 S5 Layer 1)	
163	F163	3D-Laws-114(W5 E5 W5 Layer 1)	
164	F164	3D-Laws-115(W5 L5 E5 Layer 1)	
165	F165	3D-Laws-116(W5 L5 L5 Layer 1)	
166	F166	3D-Laws-117(W5 L5 R5 Layer 1)	
167	F167	3D-Laws-118(W5 L5 S5 Layer 1)	
168	F168	3D-Laws-119(W5 L5 W5 Layer 1)	
169	F169	3D-Laws-120(W5 R5 E5 Layer 1)	
170	F170	3D-Laws-121(W5 R5 L5 Layer 1)	
171	F171	3D-Laws-122(W5 R5 R5 Layer 1)	
172	F172	3D-Laws-123(W5 R5 S5 Layer 1)	
173	F173	3D-Laws-124(W5 S5 E5 Layer 1)	
174	F174	3D-Laws-125(W5 S5 L5 Layer 1)	
175	F175	3D-Laws-126(W5 R5 W5 Layer 1)	
176	F176	3D-Laws-127(W5 S5 R5 Layer 1)	
177	F177	3D-Laws-128(W5 S5 S5 Layer 1)	
178	F178	3D-Laws-129(W5 S5 W5 Layer 1)	
179	F179	3D-Laws-130(W5 W5 E5 Layer 1)	
180	F180	3D-Laws-131(W5 W5 L5 Layer 1)	
181	F181	3D-Laws-132(W5 W5 R5 Layer 1)	
182	F182	3D-Laws-133(W5 W5 S5 Layer 1)	
183	F183	3D-Laws-134(W5 W5 W5 Layer 1)	
190	F190	3D-Wave-1(P2 L2 C9 Layer 1)	C3.Texture:<br>Laws & Wavelet Feature<br><br>(with different Wavelets)
191	F191	3D-Wave-2(P1 L2 C9 Layer 1)	
192	F192	3D-Wave-3(P2 L2 C10 Layer 1)	
193	F193	3D-Wave-4(P2 L2 C11 Layer 1)	
194	F194	3D-Wave-5(P2 L2 C12 Layer 1)	
195	F195	3D-Wave-6(P2 L2 C13 Layer 1)	
196	F196	3D-Wave-7(P2 L2 C14 Layer 1)	
197	F197	3D-Wave-8(P2 L2 C15 Layer 1)	
198	F198	3D-Wave-9(P2 L2 C1 Layer 1)	

199	F199	3D-Wave-10(P2 L2 C2 Layer 1)	
200	F200	3D-Wave-11(P2 L2 C3 Layer 1)	
201	F201	3D-Wave-12(P2 L2 C4 Layer 1)	
202	F202	3D-Wave-13(P2 L2 C5 Layer 1)	
203	F203	3D-Wave-14(P2 L2 C6 Layer 1)	
204	F204	3D-Wave-15(P2 L2 C7 Layer 1)	
205	F205	3D-Wave-16(P2 L2 C8 Layer 1)	
206	F206	3D-Wave-17(P1 L2 C11 Layer 1)	
207	F207	3D-Wave-18(P1 L2 C10 Layer 1)	
208	F208	3D-Wave-19(P1 L2 C12 Layer 1)	
209	F209	3D-Wave-20(P1 L2 C13 Layer 1)	
210	F210	3D-Wave-21(P1 L2 C14 Layer 1)	
211	F211	3D-Wave-22(P1 L2 C15 Layer 1)	
212	F212	3D-Wave-23(P1 L2 C1 Layer 1)	
213	F213	3D-Wave-24(P1 L2 C2 Layer 1)	
214	F214	3D-Wave-25(P1 L2 C3 Layer 1)	
215	F215	3D-Wave-26(P1 L2 C4 Layer 1)	
216	F216	3D-Wave-27(P1 L2 C5 Layer 1)	
217	F217	3D-Wave-28(P1 L2 C6 Layer 1)	
218	F218	3D-Wave-29(P1 L2 C7 Layer 1)	
219	F219	3D-Wave-30(P1 L2 C8 Layer 1)	
**See description (2)*			

Supplemental S.4 *(continued).* Description of Texture Features

A. **Run-length analysis:** Run-length texture features (3) examine runs of similar gray values in an image. Runs may be labeled according to their length, gray value, and direction (either horizontal or vertical). Long runs of the same gray value correspond to coarser textures, whereas shorter runs correspond to finer textures. In our study, texture information was quantified by computing 11 features (4) derived from the run-length distribution matrix. They are: 1: Short Run Emphasis (SRE). 2: Long Run Emphasis (LRE). 3: Gray-Level Non-uniformity (GLN). 4: Run Length Non-uniformity (RLN). 5: Run Percentage (RP). 6: Low Gray-Level Run Emphasis (LGRE). 7: High Gray-Level Run Emphasis (HGRE). 8: Short Run Low Gray-Level Emphasis (SRLGE). 9: Short Run High Gray-Level Emphasis (SRHGE). 10: Long Run Low Gray-Level Emphasis (LRLGE). 11: Long Run High Gray-Level Emphasis (LGHGE).

   The Co-occurrence matrices and run-length analysis features can be obtained in 3D (5), the features are calculated in 13 different directions, in each direction, processing is done by plane instead of slice. Hence, information between slices is used.

B. **Co-occurrence matrices:** the co-occurrence matrix (6) is a matrix that contains the frequency of one gray level intensity appearing in a specified spatial linear relationship with another gray level intensity within a certain range. Computation of features requires first constructing the co-occurrence matrix, then different

measurements (7) can be calculated based on the matrix. The measurements include: contrast, energy, homogeneity, entropy, mean and max probability.

C. **Laws features :** Laws features (8) were constructed from a set of five one-dimensional filters, each designed to reflect a different type of structure in the image. These one-dimensional filters are defined as E5 (edges), S5 (spots), R5 (ripples), W5 (waves), and L5 (low pass, or average gray value). By using these 1-D convolution filters, 2-D filters are generated by convolving pairs of these filters, such as L5L5, E5L5, S5L5, W5L5, R5L5, etc. We can generate 25 different 2-D filters. 3D laws filters were constructed similarly to 2D. 3D filters are generated by convolving 3 types of 1D filter, such as L5L5L5, L5L5E5, L5L5S5, L5L5R5, L5L5W5, etc. The total number of 3-D filters is 125. For the 3D case, after the convolution with the 3D filters for the image, the energy (9) of the texture feature was computed by the following equation:

$$Energy = \frac{1}{R}\sum_{i=N+1}^{I-N}\sum_{j=N+1}^{J-N}\sum_{k=N+1}^{K-N} h^2(i,j,k)\ ,$$

where R is a normalizing factor, I and J, K are image dimensions, h(i,j,k) is derived from the convolution filters and original image. For the 2D case, the above equation is very similar, but without the 3rd (z direction) dimension.

D. Wavelet Decomposition: the discrete wavelet transform (10) can iteratively decompose an image (2D) into four components. Each iteration splits the image both horizontally and vertically into low-frequency (low pass) and high-frequency (high pass) components. Thus, four components are generated: a high-pass/high-pass

component consisting of mostly diagonal structure, a high-pass/low-pass component consisting mostly of vertical structures, a low-pass/high-pass component consisting mostly of horizontal structure, and a low-pass/low-pass component that represents a blurred version of the original image. Subsequent iterations then repeat the decomposition on the low-pass/low-pass component from the previous iteration. These subsequent iterations highlight broader diagonal, vertical, and horizontal textures. And for each component, we calculated the energy (referred to with a suffix P1) & entropy (referred to with a suffix P2) feature. A wavelet transform of a 3D signal can be achieved by applying the 1D wavelet transform along all the three directions (x,y,z). Features obtained at each level of decomposition are referred with suffix L (example: L1, L2) and level of decomposition is referred to with a prefix C (example: C1 to C9).

E. **Pixel Histogram Features:** the pixel intensity histogram h(a) is the number of pixels that occurred for brightness level “a” with brightness level on the x-axis. The probability distribution of the brightness P(a) can be calculated as well. Six features: mean, standard deviation, skewness, kurtosis, energy, and entropy were then incorporated.

Supplemental Table S5. CT scanner details for reconstruction kernel variation study.

Sample size to study Scanner Variations on predictions				
**Scanner Type & Slice**	**Types**	**Total**	**Cancer**	**Normal**
Siemens 30F , 2mm<br>Siemens 50F, 2mm	Paired	95	43	52
GE Bone, 2.5 mm<br>GE Standard, 2.5 mm	Paired	53	28	25
**All Samples** (Siemens & GE)		148	71	77

Supplemental Table S6.Feature distribution for a range of difference in the Area under the Receiver operative characteristics (AUC) with different reconstruction kernels a) Number features for range of AUC difference, b) features at different AUC difference. Details of the analysis will be reported in our following study (11).

a. Number of Features (% in category)					
**Features**	Difference in test AUC: Cancer Vs Normal Discrimination				
	**Difference Range (%)**	**Train**:Siemens, B30F, 2mm<br>**Test**: Siemens, 50F, 2mm	**Train**: GE, Standard, 2.5mm<br>**Test**: GE, Bone, 2.5mm	**Train**: Siemens B30F & 50F 2mm, GE Bone & Standard, 2.5mm<br>**Test**: Siemens B30F & 50F, 2mm	**Test**: GE Bone & Standard, 2.5mm
**Size & Shape** (17 features)	≥ 0, ≤ 5%	11 (64.71)	8 (47.06)	16 (94.12)	12 (70.59)
	> 5, ≤ 10%	2 (11.76)	6 (35.29)	0 (0)	3 (17.65)
	>10, ≤15%	0 (0)	3 (17.65)	1 (5.88)	0 (0)
	>15, ≤20%	2 (11.76)	0 (0)	0 (0)	1 (5.88)
	>20, ≤30%	0 (0)	0 (0)	0 (0)	0 (0)
	>30, ≤50%	2 (11.76)	0 (0)	0 (0)	1 (5.88)
	>50%	0 (0)	0 (0)	0 (0)	0 (0)
**Location, RunL, Co-occ & Histogram** (26 features)	≥ 0, ≤ 5%	12 (46.15)	11 (42.31)	16 (61.54)	8 (30.77)
	> 5, ≤ 10%	3 (11.54)	10 (38.46)	3 (11.54)	9 (34.62)
	>10, ≤15%	3 (11.54)	5 (19.23)	7 (26.92)	5 (19.23)
	>15, ≤20%	2 (7.69)	0 (0)	0 (0)	2 (7.69)
	>20, ≤30%	1 (3.85)	0 (0)	0 (0)	1 (3.85)
	>30, ≤50%	2 (7.69)	0 (0)	0 (0)	1 (3.85)
	>50%	3 (11.54)	0 (0)	0 (0)	0 (0)
**Texture: Laws & Wavelets** (86 features)	≥ 0, ≤ 5%	84 (97.67)	83 (96.51)	84 (97.67)	81 (94.19)
	> 5, ≤ 10%	0 (0)	2 (2.33)	1 (1.16)	3 (3.49)
	>10, ≤15%	0 (0)	1 (1.16)	1 (1.16)	1 (1.16)
	>15, ≤20%	0 (0)	0 (0)	0 (0)	0 (0)
	>20, ≤30%	0 (0)	0 (0)	0 (0)	1 (1.16)
	>30, ≤50%	0 (0)	0 (0)	0 (0)	0 (0)
	>50%	2 (2.33)	0 (0)	0 (0)	0 (0)

Supplemental Table 6b. Feature with different AUC variability

**Features with Minimal Variability between Scanner Reconstructions**				
**Category**	**AUC Difference Range (%)**	**Matched Pair (difference in convolutional kernel)**		
		Siemens 30F Vs 50F (2 mm)		GE Standard Vs Bone (2.5mm)
**Size & Shape** (17 features)	[0 to ≤5]	F30:Shape-Index, F26:EllipticFit, F1:LongDia, F38:Length-Pxl, F6:Vol-cm, F36:Width-Pxl F34:Volume-pxl, F37:Thickness-Pxl, F2:ShortAx-LongDia, F41:Border-Leng-Pxl, F14:9c-3D-Compact,F25:Density.		F36:Width-Pxl,F30:Shape-Index, F34:Volume-pxl,F41:Border-Leng-Pxl, F14:9c-3D-Compact,F25:Density, F38:Length-Pxl,F6:Vol-cm
	[>5 to ≤10]	F3:ShortAx,F40:Length-by-Width,F32:RectangularFit, F39:Length-by-Thick, F23:Asymmetry.		F37:Thickness-Pxl,F26:EllipticFit, F3:ShortAx,F1:LongDia,F2:ShortAx-LongDia,F32:RectangularFit
**Location, RunL, Co-occ & Histogram** (26 features)	[0 to ≤5]	F9:8b-3D-Bord-to-Lung, F51:AvgLRE, F186:Hist-Energy-L1,F187:Hist-Entropy-L1, F21:10c-3D-Av-Vol-AirSpaces, F16:9e-3D-SD-Dist-COG-to-Border,F55:AvgRP,F48:AvgGLN, F19:10a-3D-Relat-Vol-Airspaces, F52:AvgLRHGE,F4:Mn-Hu, F17:9f-3D-Min-Dist-COG-to-Border,F189:Hist-Skew-L1		F44:AvgCooC-Constrast, F51:AvgLRE,F186:Hist-Energy-L1, F17:9f-3D-Min-Dist-COG-to-Border, F4:Mn-Hu,F20:10b-3D-Num-AirSpaces, F188:Hist-Kurt-L1,F187:Hist-Entropy-L1, F55:AvgRP,F22:10d-3D-SD-Vol-AirSpaces,F189:Hist-Skew-L1
	[>5 to ≤10]	F45:AvgCooC-Energy,F43:AvgCooc-Mp, F42:AvgCoOc-Homo,F5:Std-Hu, F188:Hist-Kurt-L1,F185:Hist-SD-L1,F8:8a-3D-Attch-Pleural		F48:AvgGLN,F19:10a-3D-Relat-Vol-Airspaces,F42:AvgCoOc-Homo, F27:Main-Direction,F5:Std-Hu, F43:AvgCooc-Mp,F8:8a-3D-Attch-Pleural,F9:8b-3D-Bord-to-Lung, F16:9e-3D-SD-Dist-COG-to-Border, F45:AvgCooC-Energy.
**Texture: Wavelets** (86 features)	[0 to ≤5]	F216:3D-WaveP1-L2-27, F215:3D-WaveP1-L2-26, F214:3D-WaveP1-L2-25 *(refer to Supplemental Table S4)*		F214:3D-WaveP1-L2-25,F216:3D-WaveP1-L2-27 *(refer to Supplemental Table S4)*
	[>5 to ≤10]	F60:3D-Laws-2 *(for additional features refer to Supplemental Table S4)*		F215:3D-WaveP1-L2-26,F60:3D-Laws-2 *(for additional features refer to Supplemental Table S4)*

Supplemental Table S7. Summary of Area under the Receiver operative characteristics (AUC) between training on a CT images with consistent convolution kernel type to testing on an image with different reconstruction kernel. In the summary tables, a) training with Siemens B30F, 2mm b) training with GE Standard, 2.5mm, c) training with mixed scans & slice thickness d) Features for difference AUC difference range.

**Features**	**(a) Difference in test AUC: Cancer Vs Normal Discrimination (Training: CT on Siemens B30F, 2mm (D1))**								
	Difference Range	Δ(D1 Vs 30F, 5mm) (%)	Δ(D1 Vs 50F, 2mm) (%)	Δ(D1 Vs 50F, 5mm) (%)	Δ(D1 Vs All Siemens) (%)	Δ(D1 Vs GE Standard, 2.5mm) (%)	Δ(D1 Vs GE Bone, 2.5mm) (%)	{Δ(D1 Vs All GE) (%)	{Δ(D1 Vs All) (%)
**Size & Shape** (17 features)	≥ 0, ≤ 5%	12 (70.59)	11 (64.71)	10 (58.82)	13 (76.47)	6 (35.29)	3 (17.65)	10 (58.82)	13 (76.47)
	> 5, ≤ 10%	5 (29.41)	2 (11.76)	3 (17.65)	1 (5.88)	5 (29.41)	7 (41.18)	3 (17.65)	2 (11.76)
	>10, ≤15%	0 (0)	0 (0)	0 (0)	1 (5.88)	1 (5.88)	3 (17.65)	1 (5.88)	1 (5.88)
	>15, ≤20%	0 (0)	2 (11.76)	0 (0)	1 (5.88)	2 (11.76)	2 (11.76)	1 (5.88)	1 (5.88)
	>20, ≤30%	0 (0)	0 (0)	1 (5.88)	1 (5.88)	1 (5.88)	2 (11.76)	2 (11.76)	0 (0)
	>30, ≤50%	0 (0)	2 (11.76)	2 (11.76)	0 (0)	2 (11.76)	0 (0)	0 (0)	0 (0)
	>50%	0 (0)	0 (0)	1 (5.88)	0 (0)	0 (0)	0 (0)	0 (0)	0 (0)
**Location, RunL, CoOcc & Histogram** (26 features)	≥ 0, ≤ 5%	13 (50)	12 (46.15)	9 (34.62)	16 (61.54)	7 (26.92)	8 (30.77)	6 (23.08)	11 (42.31)
	> 5, ≤ 10%	7 (26.92)	3 (11.54)	5 (19.23)	3 (11.54)	4 (15.38)	5 (19.23)	9 (34.62)	4 (15.38)
	>10, ≤15%	3 (11.54)	3 (11.54)	4 (15.38)	2 (7.69)	8 (30.77)	7 (26.92)	3 (11.54)	9 (34.62)
	>15, ≤20%	1 (3.85)	2 (7.69)	1 (3.85)	1 (3.85)	2 (7.69)	1 (3.85)	3 (11.54)	1 (3.85)
	>20, ≤30%	1 (3.85)	1 (3.85)	3 (11.54)	4 (15.38)	4 (15.38)	5 (19.23)	5 (19.23)	1 (3.85)
	>30, ≤50%	1 (3.85)	2 (7.69)	1 (3.85)	0 (0)	1 (3.85)	0 (0)	0 (0)	0 (0)
	>50%	0 (0)	3 (11.54)	3 (11.54)	0 (0)	0 (0)	0 (0)	0 (0)	0 (0)
**Texture: Laws & Wavelets** (86 features)	≥ 0, ≤ 5%	81 (94.19)	84 (97.67)	82 (95.35)	84 (97.67)	83 (96.51)	82 (95.35)	82 (95.35)	84 (97.67)
	> 5, ≤ 10%	3 (3.49)	0 (0)	2 (2.33)	0 (0)	2 (2.33)	1 (1.16)	2 (2.33)	0 (0)
	>10, ≤15%	1 (1.16)	0 (0)	0 (0)	0 (0)	1 (1.16)	2 (2.33)	2 (2.33)	0 (0)
	>15, ≤20%	1 (1.163)	0 (0)	0 (0)	0 (0)	0 (0)	1 (1.16)	0 (0)	2 (2.33)
	>20, ≤30%	0 (0)	0 (0)	0 (0)	2 (2.33)	0 (0)	0 (0)	0 (0)	0 (0)
	>30, ≤50%	0 (0)	0 (0)	0 (0)	0 (0)	0 (0)	0 (0)	0 (0)	0 (0)
	>50%	0 (0)	2 (2.33)	2 (2.33)	0 (0)	0 (0)	0 (0)	0 (0)	0 (0)

Features Category	(b) Difference in test AUC: Cancer Vs Normal Discrimination (Training: GE Standard, 2.5 mm (D2))							
	Difference Range	Δ(D2 Vs 30F, 2mm)	Δ(D2 Vs 30F, 5mm)	Δ(D2 Vs 50F, 2mm)	Δ(D2 Vs 50F, 5mm)	Δ(D2 Vs All Siemens)	Δ(D2 Vs GE Bone, 2.5mm)	{Δ(D2 Vs All GE)	{Δ(D2 Vs All)
**Size & Shape** (17 features)	≥ 0, ≤ 5%	6 (35.29)	4 (23.53)	4 (23.53)	3 (17.65)	6 (35.29)	8 (47.06)	12 (70.59)	9 (52.94)
	> 5, ≤ 10%	5 (29.41)	6 (35.29)	6 (35.29)	6 (35.29)	6 (35.29)	6 (35.29)	1 (5.88)	3 (17.65)
	>10, ≤15%	0 (0)	3 (17.65)	3 (17.65)	3 (17.65)	0 (0)	3 (17.65)	3 (17.65)	1 (5.88)
	>15, ≤20%	3 (17.65)	0 (0)	1 (5.88)	0 (0)	2 (11.76)	0 (0)	1 (5.88)	2 (11.76)
	>20, ≤30%	2 (11.76)	2 (11.76)	2 (11.76)	2 (11.76)	2 (11.76)	0 (0)	0 (0)	1 (5.88)
	>30, ≤50%	1 (5.88)	2 (11.76)	0 (0)	2 (11.76)	1 (5.88)	0 (0)	0 (0)	1 (5.88)
	>50%	0 (0)	0 (0)	1 (5.88)	1 (5.88)	0 (0)	0 (0)	0 (0)	0 (0)
**Location, RunL, CoOcc & Histogram** (26 features)	≥ 0, ≤ 5%	7 (26.92%)	3 (11.54%)	7 (26.92%)	3 (11.54%)	5 (19.23%)	11 (42.31%)	11 (42.31%)	6 (23.08%)
	> 5, ≤ 10%	4 (15.38%)	3 (11.54%)	5 (19.23%)	11 (42.31%)	5 (19.23%)	10 (38.46%)	14 (53.85%)	9 (34.62%)
	>10, ≤15%	3 (11.54%)	5 (19.23%)	5 (19.23%)	5 (19.23%)	3 (11.54%)	5 (19.23%)	1 (3.85%)	7 (26.92%)
	>15, ≤20%	8 (30.77%)	3 (11.54%)	2 (7.69%)	1 (3.85%)	8 (30.77%)	0 (0%)	0 (0%)	3 (11.54%)
	>20, ≤30%	4 (15.38%)	10 (38.46%)	3 (11.54%)	2 (7.69%)	4 (15.38%)	0 (0%)	0 (0%)	0 (0%)
	>30, ≤50%	0 (0%)	2 (7.69%)	1 (3.85%)	1 (3.85%)	1 (3.85%)	0 (0%)	0 (0%)	1 (3.85%)
	>50%	0 (0%)	0 (0%)	3 (11.54%)	3 (11.54%)	0 (0%)	0 (0%)	0 (0%)	0 (0%)
**Texture: Laws & Wavelets** (86 features)	≥ 0, ≤ 5%	83 (96.51)	81 (94.19)	81 (94.19)	82 (95.35)	81 (94.19)	83 (96.51)	86 (100)	81 (94.19)
	> 5, ≤ 10%	1 (1.16)	1 (1.16)	3 (3.49)	1 (1.16)	0 (0)	2 (2.33)	0 (0)	3 (3.49)
	>10, ≤15%	2 (2.33)	2 (2.33)	0 (0)	1 (1.16)	3 (3.49)	1 (1.16)	0 (0)	1 (1.16)
	>15, ≤20%	0 (0)	1 (1.16)	0 (0)	0 (0)	0 (0)	0 (0)	0 (0)	1 (1.16)
	>20, ≤30%	0 (0)	1 (1.16)	0 (0)	0 (0)	2 (2.33)	0 (0)	0 (0)	0 (0)
	>30, ≤50%	0 (0)	0 (0)	0 (0)	1 (1.16)	0 (0)	0 (0)	0 (0)	0 (0)
	>50%	0 (0)	0 (0)	2 (2.33)	1 (1.16)	0 (0)	0 (0)	0 (0)	0 (0)

**Features Category**	**(c)Difference in test AUC: Cancer Vs Normal Discrimination (Training: All available – Siemens & GE, (D3))**							
	Difference Range	Δ(D3 Vs 30F, 2mm)	Δ(D3 Vs 30F, 5mm)	Δ(D3 Vs 50F, 2mm)	Δ(D3 Vs 50F, 5mm)	Δ(D3 Vs All Siemens)	Δ(D3 Vs GE Standard, 2.5mm)	Δ(D3 Vs GE Bone, 2.5mm)	{Δ(D3 Vs All GE)
**Size & Shape** (17 features)	≥ 0, ≤ 5%	13 (76.47)	14 (82.35)	12 (70.59)	11 (64.71)	16 (94.12)	10 (58.82)	4 (23.53)	12 (70.59)
	> 5, ≤ 10%	2 (11.76)	2 (11.76)	1 (5.88)	2 (11.76)	0 (0)	2 (11.76)	8 (47.06)	3 (17.65)
	>10, ≤15%	1 (5.88)	1 (5.88)	1 (5.88)	0 (0)	1 (5.88)	0 (0)	2 (11.76)	0 (0)
	>15, ≤20%	1 (5.88)	0 (0)	1 (5.88)	1 (5.88)	0 (0)	2 (11.76)	2 (11.76)	1 (5.88)
	>20, ≤30%	0 (0)	0 (0)	1 (5.88)	1 (5.88)	0 (0)	1 (5.88)	0 (0)	0 (0)
	>30, ≤50%	0 (0)	0 (0)	1 (5.88)	2 (11.76)	0 (0)	1 (5.88)	1 (5.88)	1 (5.88)
	>50%	0 (0)	0 (0)	0 (0)	0 (0)	0 (0)	1 (5.88)	0 (0)	0 (0)
**Location, RunL, CoOcc & Histogram** (26 features)	≥ 0, ≤ 5%	11 (42.31)	11 (42.31)	11 (42.31)	14 (53.85)	16 (61.54)	7 (26.92)	11 (42.31)	8 (30.77)
	> 5, ≤ 10%	4 (15.39)	8 (30.77)	4 (15.39)	4 (15.39)	3 (11.54)	6 (23.08)	5 (19.23)	9 (34.62)
	>10, ≤15%	10 (38.46)	2 (7.69)	5 (19.23)	2 (7.69)	7 (26.92)	10 (38.46)	7 (26.92)	5 (19.23)
	>15, ≤20%	0 (0)	3 (11.54)	1 (3.85)	1 (3.85)	0 (0)	1 (3.85)	1 (3.85)	2 (7.69)
	>20, ≤30%	1 (3.85)	2 (7.69)	2 (7.69)	2 (7.69)	0 (0)	1 (3.85)	1 (3.85)	1 (3.85)
	>30, ≤50%	0 (0)	0 (0)	1 (3.85)	1 (3.85)	0 (0)	1 (3.85)	1 (3.85)	1 (3.85)
	>50%	0 (0)	0 (0)	2 (7.69)	2 (7.69)	0 (0)	0 (0)	0 (0)	0 (0)
**Texture: Laws & Wavelets** (86 features)	≥ 0, ≤ 5%	84 (97.67)	82 (95.35)	84 (97.67)	84 (97.67)	84 (97.67)	81 (94.19)	81 (94.19)	81 (94.19)
	> 5, ≤ 10%	0 (0)	2 (2.33)	0 (0)	0 (0)	1 (1.16)	3 (3.49)	0 (0)	3 (3.49)
	>10, ≤15%	0 (0)	2 (2.33)	0 (0)	0 (0)	1 (1.16)	0 (0)	3 (3.49)	1 (1.16)
	>15, ≤20%	0 (0)	0 (0)	0 (0)	0 (0)	0 (0)	2 (2.33)	0 (0)	0 (0)
	>20, ≤30%	2 (2.33)	0 (0)	0 (0)	0 (0)	0 (0)	0 (0)	1 (1.16)	1 (1.16)
	>30, ≤50%	0 (0)	0 (0)	1 (1.16)	2 (2.33)	0 (0)	0 (0)	1 (1.16)	0 (0)
	>50%	0 (0)	0 (0)	1 (1.16)	0 (0)	0 (0)	0 (0)	0 (0)	0 (0)

See Attached excel sheet for details, three worksheets corresponds to a),b) & c) (SupplementalTableS5_ExcelSheetAttach_forREconAUC.xlsx)

(Picture shown for reference).

GID-1	Catg3	CategoryDesc	Error-HoldOut(train:	DeltaAUC	DeltaAUC	DeltaAUC	DeltaAUC	DeltaAUC	DeltaAUC	DeltaAUC	DeltaAUC
F1:LongDia	1	Size&Shape	0.251052	0.011882	0.000276	0.006986	0.002493	0.00806	0.069634	0.030601	0.002609
F2:ShortAx-LongDia	1	Size&Shape	0.25421	0.026203	0.001301	0.007758	0.014031	0.000719	0.072462	0.005157	0.013482
F3:ShortAx	1	Size&Shape	0.251579	0.050865	0.011846	0.057256	0.030358	0.002989	0.05322	0.026108	0.034412
F6:Vol-cm	1	Size&Shape	0.323421	0.015049	0.007617	0.015458	0.007618	0.062601	0.10487	0.050027	0.019125
F14:9c-3D-Compact	1	Size&Shape	0.320526	0.033137	0.045698	0.036644	0.018729	0.064421	0.086615	0.033082	0.008425
F23:Asymmetry	1	Size&Shape	0.522105	0.099831	0.1859	0.072061	0.016957	0.394459	0.202585	0.20183	0.022916
F25:Density	1	Size&Shape	0.390789	0.042479	0.061661	0.079356	0.014618	0.21899	0.172276	0.01236	0.010744
F26:EllipticFit	1	Size&Shape	0.327105	0.004717	0.064888	0.224693	0.050632	0.131742	0.178827	0.126138	0.060932
F30:Shape-Index	1	Size&Shape	0.20421	0.003737	0.007423	0.010665	0.018651	0.065421	0.077359	0.054947	0.031212
F32:RectangularFit	1	Size&Shape	0.313947	0.098975	0.353209	0.378504	0.156432	0.173265	0.100584	0.150655	0.150989
F34:Volume-pxl	1	Size&Shape	0.311315	0.021356	0.01971	0.033275	0.00823	0.051777	0.070537	0.026877	0.002561
F36:Width-Pxl	1	Size&Shape	0.231579	0.020001	0.035822	0.039255	0.016835	0.031354	0.039987	0.012991	0.005954
F37:Thickness-Pxl	1	Size&Shape	0.219737	0.021522	0.017427	0.020365	0.013559	0.161605	0.116332	0.077418	0.00793
F38:Length-Pxl	1	Size&Shape	0.231842	0.012767	0.003524	0.026886	0.003639	0.006034	0.045112	0.025575	0.006407
F39:Length-by-Thick	1	Size&Shape	0.518158	0.099633	0.492261	0.587099	0.24726	0.462753	0.255445	0.25463	0.130097
F40:Length-by-Width	1	Size&Shape	0.402105	0.061396	0.197231	0.356485	0.108498	0.07375	0.048823	0.006717	0.071924
F41:Border-Leng-Pxl	1	Size&Shape	0.248684	0.031032	0.024158	0.021892	0.016635	0.041201	0.061711	0.022494	0.005118
F4:Mn-Hu	2	Location,Runl,CoOc,Histog	0.484474	0.029247	0.022321	0.001396	0.008131	0.216834	0.204906	0.233714	0.114302
F5:Std-Hu	2	Location,Runl,CoOc,Histog	0.523947	0.068391	0.304199	0.258283	0.102381	0.203426	0.126167	0.141743	0.106379
F8:8a-3D-Attch-Pleural	2	Location,Runl,CoOc,Histog	0.468684	0.097856	0.088984	0.143782	0.083896	0.058377	0.126835	0.120437	0.090831
F9:8b-3D-Bord-to-Lung	2	Location,Runl,CoOc,Histog	0.519737	0	0	0	0	0	0	0	0
F12:9a-3D-FractionalAr	2	Location,Runl,CoOc,Histog	0.513684	0.113079	0.506827	0.61404	0.255217	0.363974	0.202238	0.187905	0.148089
F16:9e-3D-SD-Dist-COC	2	Location,Runl,CoOc,Histog	0.279473	0.013379	0.147838	0.039252	0.067183	0.061201	0.024266	0.002641	0.051372
F17:9f-3D-Min-Dist-CO	2	Location,Runl,CoOc,Histog	0.269473	0.035675	0.205066	0.281802	0.051238	0.207759	0.206854	0.164989	0.057687
F19:10a-3D-Relat-Vol-/	2	Location,Runl,CoOc,Histog	0.438421	0.022625	0.02381	0.011777	0.022077	0.18749	0.141031	0.204742	0.082249
F20:10b-3D-Num-AirSp	2	Location,Runl,CoOc,Histog	0.432368	0.366828	0.194019	0.247731	0.203267	0.179547	0.209154	0.205256	0.270521
F21:10c-3D-Av-Vol-Air!	2	Location,Runl,CoOc,Histog	0.446579	0.013327	0.036036	0.108599	0.016287	0.231495	0.080979	0.26162	0.125037
F22:10d-3D-SD-Vol-Air	2	Location,Runl,CoOc,Histog	0.450789	0.196736	0.047799	0.156945	0.119111	0.046925	0.08809	0.110334	0.14486
F27:Main-Direction	2	Location,Runl,CoOc,Histog	0.50579	0.209447	0.44785	0.495676	0.244101	0.078444	0.010103	0.052459	0.17178
F42:AvgCoOc-Homo	2	Location,Runl,CoOc,Histog	0.198158	0.065374	0.033375	0.059183	0.006297	0.129314	0.077992	0.076501	0.010221
F43:AvgCooc-Mp	2	Location,Runl,CoOc,Histog	0.21421	0.063269	0.030206	0.056288	0.006823	0.137937	0.075339	0.078527	0.010494
F44:AvgCooC-Constras	2	Location,Runl,CoOc,Histog	0.362105	0.112467	0.599922	0.561819	0.194334	0.006405	0.006405	0.035579	0.104231
F45:AvgCooC-Energy	2	Location,Runl,CoOc,Histog	0.24	0.058361	0.042287	0.066052	0.000252	0.141379	0.066092	0.078871	0.016604
F47:AvgCoOc-Mean	2	Location,Runl,CoOc,Histog	0.435263	0.109865	0.604368	0.585377	0.20049	0.028344	0.105348	0.056271	0.115099
F48:AvgGLN	2	Location,Runl,CoOc,Histog	0.353421	0.021943	0.013196	0.019992	0.012016	0.077095	0.123825	0.057396	0.005424
F51:AvgLRE	2	Location,Runl,CoOc,Histog	0.202631	0	0	0	0	0	0	0	0
F52:AvgLRHGE	2	Location,Runl,CoOc,Histog	0.348684	0.023498	0.076736	0.068443	0.014227	0.147217	0.238959	0.210447	0.111219
F55:AvgRP	2	Location,Runl,CoOc,Histog	0.232631	0.01845	0.015738	0.006809	0.010459	0.110921	0.142914	0.070215	0.007374
F185:Hist-SD-L1	2	Location,Runl,CoOc,Histog	0.352631	0.089286	0.15303	0.110178	0.020804	0.10636	0.005589	0.057586	0.026859
F186:Hist-Energy-L1	2	Location,Runl,CoOc,Histog	0.292894	0	0	0	0	0	0	0	0
F187:Hist-Entropy-L1	2	Location,Runl,CoOc,Histog	0.239473	0.009735	0.051268	0.035237	0.021318	0.132757	0.103388	0.063247	0.001186
F188:Hist-Kurt-L1	2	Location,Runl,CoOc,Histog	0.45421	0.080238	0.13225	0.121017	0.000912	0.015025	0.01463	0.015003	0.000388
F189:Hist-Skew-L1	2	Location,Runl,CoOc,Histog	0.506053	0.047678	0.131245	0.096708	0.016077	0.140308	0.178383	0.183487	0.104507
F59:3D-Laws-1	3	Texture:Laws&Wavelet	0.534474	0	0	0	0	0	0	0	0
F60:3D-Laws-2	3	Texture:Laws&Wavelet	0.384473	0.108681	0.575153	0.583323	0.252406	0.028362	0.04559	0.052216	0.171183
F62:3D-Laws-4	3	Texture:Laws&Wavelet	0.535	0	0	0	0	0	0	0	0
F63:3D-Laws-5	3	Texture:Laws&Wavelet	0.46	0	0	0	0	0	0	0	0
F64:3D-Laws-6	3	Texture:Laws&Wavelet	0.364473	0	0	0	0	0	0	0	0
F67:3D-Laws-9	3	Texture:Laws&Wavelet	0.406842	0	0	0	0	0	0	0	0
F68:3D-Laws-10	3	Texture:Laws&Wavelet	0.397368	0	0	0	0	0	0	0	0
F69:3D-Laws-11	3	Texture:Laws&Wavelet	0.519474	0	0	0	0	0	0	0	0
F72:3D-Laws-14	3	Texture:Laws&Wavelet	0.525263	0	0	0	0	0	0	0	0
F73:3D-Laws-15	3	Texture:Laws&Wavelet	0.527105	0	0	0	0	0	0	0	0
F74:3D-Laws-16	3	Texture:Laws&Wavelet	0.44421	0	0	0	0	0	0	0	0
F75:3D-Laws-17	3	Texture:Laws&Wavelet	0.520526	0	0	0	0	0	0	0	0
F76:3D-Laws-18	3	Texture:Laws&Wavelet	0.321842	0	0	0	0	0	0	0	0
F77:3D-Laws-19	3	Texture:Laws&Wavelet	0.481316	0	0	0	0	0	0	0	0
F78:3D-Laws-20	3	Texture:Laws&Wavelet	0.382631	0	0	0	0	0	0	0	0
F79:3D-Laws-21	3	Texture:Laws&Wavelet	0.537368	0	0	0	0	0	0	0	0
F80:3D-Laws-22	3	Texture:Laws&Wavelet	0.416842	0	0	0	0	0	0	0	0
F82:3D-Laws-24	3	Texture:Laws&Wavelet	0.51	0	0	0	0	0	0	0	0
F83:3D-Laws-25	3	Texture:Laws&Wavelet	0.506316	0	0	0	0	0	0	0	0

Key for Abbreviations Used in the Classifier Results Section:

Error: Classifier error (1- Accuracy)
Sensitivity(or Recall): True Positive Rate (TPR) =>(True Positive/True Positive + False Negative)
Specificity: True Negative Rate (TNR) => (True Negative/True Negative + False Positive)
PPV (or Precision): Positive predictive value: => (True Positive/True Positive + False positive)
NPV: Negative Predictive value => (True negative/True negative + False negative)
AUC: Area under the Receiver Operator Curve
CI: Confidence Limits (on AUC).

Table S8. Top images features discriminating cancer to benign nodules using 129reproducible, non-redundant features. A) performance of radiomic model with increasing feature dimension and B) four feature predictors , across all feature category types.

A)

All categories: Cancer Vs Normal (129 Reproducible, Non-Redundant Features) – Hold out CV					
	Dimensions	Feature	E[Error]	E[Sen], E[Spec], (J-index)	E[AUC], with CI
1	4 Features	F3:ShortAx, F4:Mn-Hu,,F26:EllipticFit, F150:3D-Laws-101	0.202	0.484, 0.929, (0.414)	0.834 [0.689,0.939]
2	3 Features	F3:ShortAx, F4:Mn-Hu, F26:EllipticFit	0.235	0.506, 0.928, (0.435)	0.842 [0.71, 0.93]
3	2 Features	F4:Mn-Hu, F37:Thickness-Pxl	0.271	0.462, 0.945, (0.407)	0.827 [0.694, 0.936]
4	1 Feature	F1:LongDia	0.239	0.396, 0.945, (0.340)	0.786 [0.603, 0.916]

B)

**TRAINING (4 Feature) : Primary : Cancer Vs Norm (78 Vs 166) || All**							
**Features**	**Error**	**Sensitivity**	**Specificity**	**PPV**	**NPV**	**AUC**	
						**μ (σ)**	**CI**
F19:10a-3D-Relat-Vol-Airspaces;F36:Width-Pxl;F48:AvgGLN;F192:3D-WaveP2-L2-3	0.213	0.573	0.891	0.717	0.815	0.728 (0.141)	[0.522,0.927]
F19:10a-3D-Relat-Vol-Airspaces;F34:Volume-pxl;F37:Thickness-Pxl;F48:AvgGLN	0.211	0.505	0.929	0.777	0.796	0.801 (0.07)	[0.652,0.921]
F19:10a-3D-Relat-Vol-Airspaces;F21:10c-3D-Av-Vol-AirSpaces;F32:RectangularFit;F198:3D-WaveP2-L2-9	0.211	0.538	0.911	0.746	0.806	0.818 (0.069)	[0.66,0.948]
F3:ShortAx;F4:Mn-Hu;F26:EllipticFit;F176:3D-Laws-127	0.195	0.518	0.946	0.826	0.801	0.833 (0.055)	[0.708,0.941]
F19:10a-3D-Relat-Vol-Airspaces;F21:10c-3D-Av-Vol-AirSpaces;F22:10d-3D-SD-Vol-AirSpaces;F201:3D-WaveP2-L2-12	0.207	0.605	0.891	0.735	0.817	0.825 (0.059)	[0.704,0.933]

**ImageF+Clinc (4Feature): Primary (Canc Vs Norm) || Test (88 Vs 147)|| All**							
**F1**	**Error**	**Sensitivity**	**Specificity**	**PPV**	**NPV**	**AUC**	**CI**
Comb1	0.224	0.483	0.912	0.72	0.793	0.793(0.067)	[0.634,0.937]
Comb2	0.231	0.435	0.929	0.75	0.775	0.798(0.057)	[0.678,0.892]
Comb6	0.222	0.474	0.919	0.736	0.791	0.81(0.058)	[0.676,0.932]
Comb8	0.219	0.468	0.928	0.756	0.791	0.815(0.061)	[0.683,0.936]
Comb9	0.234	0.443	0.917	0.71	0.781	0.806(0.062)	[0.668,0.916]

**Clinc (4Feature): Primary (Canc Vs Norm) || Test (88 Vs 147)|| All**							
**Clinc**	**Error**	**Sensitivity**	**Specificity**	**PPV**	**NPV**	**AUC**	**CI**
Age, Gender, Pky, Smoke-Status	0.325	0.003	0.997	0.022	0.677	0.522 (0.083)	[0.364,0.689]

a. TESTING (4F: Img): Primary (Canc Vs Norm) || Test (88 Vs 147)|| All						
**F1**	**Error**	**Sensitivity**	**Specificity**	**PPV**	**NPV**	**AUC**
F19:10a-3D-Relat-Vol-Airspaces;F36:Width-Pxl;F48:AvgGLN;F192:3D-WaveP2-L2-3	0.162	0.625	0.966	0.917	0.811	0.889
F19:10a-3D-Relat-Vol-Airspaces;F34:Volume-pxl;F37:Thickness-Pxl;F48:AvgGLN	0.17	0.568	0.986	0.962	0.792	0.9
F19:10a-3D-Relat-Vol-Airspaces;F21:10c-3D-Av-Vol-AirSpaces;F32:RectangularFit;F198:3D-WaveP2-L2-9	0.187	0.614	0.932	0.844	0.801	0.863
F3:ShortAx;F4:Mn-Hu;F26:EllipticFit;F176:3D-Laws-127	0.179	0.568	0.973	0.926	0.79	0.902
F19:10a-3D-Relat-Vol-Airspaces;F21:10c-3D-Av-Vol-AirSpaces;F22:10d-3D-SD-Vol-AirSpaces;F201:3D-WaveP2-L2-12	0.191	0.614	0.925	0.831	0.8	0.872

b. TESTING (4F: Img+Clinc): Primary (Canc Vs Norm) || Test (88 Vs 147)|| All						
**F1**	**Error**	**Sensitivity**	**Specificity**	**PPV**	**NPV**	**AUC**
Comb1	0.196	0.545	0.959	0.889	0.779	0.879
Comb2	0.187	0.523	0.986	0.958	0.775	0.89
Comb6	0.213	0.557	0.925	0.817	0.777	0.859
Comb8	0.196	0.523	0.973	0.92	0.773	0.898
Comb9	0.191	0.602	0.932	0.841	0.797	0.867

Table S9. Classification results for training and independent testing using reproducible & non-redundant features in the following category I) C1:Size & Shape features, II) C2:Location, RunL, CoOcc & Histogram features III) C3:Texture features.

I) Size & Shape Features:

a.**TRAINING (4 Feature) : Primary: Cancer Vs Norm (78 Vs 166) || C1: Size & Shape Features**							
**Features**	**Error**	**Sensitivity**	**Specificity**	**PPV**	**NPV**	**AUC**	
						**μ (σ)**	**CI**
F2:ShortAx-LongDia;F3:ShortAx;F25:Density;F32:RectangularFit	0.216	0.465	0.938	0.788	0.786	0.79 (0.069)	[0.651,0.902]
F2:ShortAx-LongDia;F3:ShortAx;F39:Length-by-Thick;F32:RectangularFit	0.224	0.465	0.931	0.775	0.778	0.791 (0.057)	[0.658,0.916]
F2:ShortAx-LongDia;F3:ShortAx;F37:Thickness-Pxl;F32:RectangularFit	0.221	0.457	0.934	0.774	0.783	0.797 (0.07)	[0.655,0.919]
F2:ShortAx-LongDia;F3:ShortAx;F41:Border-Leng-Pxl;F32:RectangularFit	0.227	0.454	0.934	0.779	0.773	0.801 (0.061)	[0.677,0.92]
F2:ShortAx-LongDia;F3:ShortAx;F38:Length-Pxl;F32:RectangularFit	0.233	0.443	0.931	0.765	0.769	0.8(0.061)	[0.662,0.91]

**b. Clinc (4Feature): Primary (Canc Vs Norm) || Test (88 Vs 147)||**							
**Clinc**	**Error**	**Sensitivity**	**Specificity**	**PPV**	**NPV**	**AUC**	**CI**
Age, Gender, Pky, Smoke-Status	0.321	0.002	0.994	0.01	0.681	0.533 (0.073)	[0.372,0.672]

**c. ImageF+Clinc (4Feature): Primary (Canc Vs Norm) || Test (88 Vs 147)|| C1: Size & Shape Features**							
**F1**	**Error**	**Sensitivity**	**Specificity**	**PPV**	**NPV**	**AUC**	**CI**
Comb1	0.221	0.478	0.915	0.722	0.797	0.789(0.075)	[0.595,0.91]
Comb2	0.232	0.434	0.919	0.713	0.783	0.774(0.077)	[0.587,0.935]
Comb3	0.236	0.454	0.918	0.738	0.774	0.783(0.054)	[0.678,0.89]
Comb4	0.233	0.466	0.917	0.736	0.779	0.792(0.07)	[0.604,0.904]
Comb6	0.223	0.452	0.924	0.736	0.789	0.783(0.066)	[0.651,0.92]

**d. TESTING (4Feature): Primary (Canc Vs Norm) || Test (88 Vs 147 || C1: Size & Shape Features**						
**Features**	**Error**	**Sensitivity**	**Specificity**	**PPV**	**NPV**	**AUC**
F2:ShortAx-LongDia;F3:ShortAx;F25:Density;F32:RectangularFit	0.179	0.557	0.98	0.942	0.787	0.858
F2:ShortAx-LongDia;F3:ShortAx;F39:Length-by-Thick;F32:RectangularFit	0.179	0.557	0.98	0.942	0.787	0.859
F2:ShortAx-LongDia;F3:ShortAx;F37:Thickness-Pxl;F32:RectangularFit	0.179	0.557	0.98	0.942	0.787	0.863
F2:ShortAx-LongDia;F3:ShortAx;F41:Border-Leng-Pxl;F32:RectangularFit	0.179	0.557	0.98	0.942	0.787	0.86
F2:ShortAx-LongDia;F3:ShortAx;F38:Length-Pxl;F32:RectangularFit	0.179	0.557	0.98	0.942	0.787	0.86

**e.TESTING (4F: Img+Clinc): Primary (Canc Vs Norm) || Test (88 Vs 147)|| C1: Size & Shape**						
**F1**	**Error**	**Sensitivity**	**Specificity**	**PPV**	**NPV**	**AUC**
Comb1	0.191	0.534	0.973	0.922	0.777	0.85
Comb2	0.2	0.523	0.966	0.902	0.772	0.85
Comb3	0.187	0.545	0.973	0.923	0.781	0.856
Comb4	0.196	0.534	0.966	0.904	0.776	0.851
Comb6	0.196	0.534	0.966	0.904	0.776	0.852

II. **Location, RunL, CoOcc & Histogram**:

**TRAINING (4 Feature) : Primary: Cancer Vs Norm (78 Vs 166) || C2: Loc, CoOc& RunL Features (Sort: AUC, Sen/Spec)**							
**Features**	**Error**	**Sensitivity**	**Specificity**	**PPV**	**NPV**	**AUC**	
						**µ (σ)**	**CI**
F27:Main-Direction;F185:Hist-SD-L1;F186:Hist-Energy-L1;F187:Hist-Entropy-L1	0.224	0.609	0.86	0.686	0.818	0.788(0.08)	[0.587,0.929]
F185:Hist-SD-L1;F186:Hist-Energy-L1;F187:Hist-Entropy-L1;F44:AvgCooC-Constrast	0.23	0.611	0.851	0.669	0.817	0.792(0.069)	[0.649,0.941]
F9:8b-3D-Bord-to-Lung;F19:10a-3D-Relat-Vol-Airspaces;F21:10c-3D-Av-Vol-AirSpaces;F42:AvgCoOc-Homo	0.215	0.544	0.908	0.746	0.802	0.851(0.053)	[0.733,0.949]
F9:8b-3D-Bord-to-Lung;F21:10c-3D-Av-Vol-AirSpaces;F42:AvgCoOc-Homo;F52:AvgLRHGE	0.198	0.507	0.941	0.807	0.802	0.809(0.06)	[0.692,0.911]
F9:8b-3D-Bord-to-Lung;F19:10a-3D-Relat-Vol-Airspaces;F21:10c-3D-Av-Vol-AirSpaces;F43:AvgCooc-Mp	0.216	0.539	0.904	0.736	0.803	0.831(0.061)	[0.688,0.931]

**Clinc (4Feature): Primary a (Canc Vs Norm) || Test (88 Vs 147)||**							
**Clinc**	**Error**	**Sensitivity**	**Specificity**	**PPV**	**NPV**	**AUC**	**CI**
Age, Gender, Pky, Smoke-Status	0.328	0.008	0.99	0.052	0.676	0.511 (0.089)	[0.308,0.651]

ImageF+Clinc (4Feature): Primary (Canc Vs Norm) || Test (88 Vs 147)|| C2: Loc, CoOc& RunL Features

F1	Error	Sensitivity	Specificity	PPV	NPV	AUC	CI
Comb1	0.252	0.544	0.848	0.63	0.796	0.768(0.068)	[0.644,0.926]
Comb2	0.25	0.531	0.859	0.642	0.794	0.774(0.061)	[0.638,0.92]
Comb4	0.218	0.533	0.909	0.745	0.799	0.844(0.061)	[0.708,0.944]
Comb7	0.227	0.477	0.915	0.733	0.785	0.801(0.067)	[0.659,0.908]
Comb9	0.222	0.513	0.908	0.74	0.795	0.819(0.062)	[0.699,0.924]

TESTING (4Feature): Primary (Canc Vs Norm) || Test (88 Vs 147 || C2: Loc, CoOc& RunL Features (Sort: AUC, Sen/Spec)

Features	Error	Sensitivity	Specificity	PPV	NPV	AUC
F27:Main-Direction;F185:Hist-SD-L1;F186:Hist-Energy-L1;F187:Hist-Entropy-L1	0.191	0.625	0.918	0.821	0.804	0.856
F185:Hist-SD-L1;F186:Hist-Energy-L1;F187:Hist-Entropy-L1;F44:AvgCooC-Constrast	0.174	0.67	0.918	0.831	0.823	0.858
F9:8b-3D-Bord-to-Lung;F19:10a-3D-Relat-Vol-Airspaces;F21:10c-3D-Av-Vol-AirSpaces;F42:AvgCoOc-Homo	0.166	0.693	0.918	0.836	0.833	0.878
F9:8b-3D-Bord-to-Lung;F21:10c-3D-Av-Vol-AirSpaces;F42:AvgCoOc-Homo;F52:AvgLRHGE	0.209	0.545	0.939	0.842	0.775	0.861
F9:8b-3D-Bord-to-Lung;F19:10a-3D-Relat-Vol-Airspaces;F21:10c-3D-Av-Vol-AirSpaces;F43:AvgCooc-Mp	0.209	0.625	0.891	0.775	0.799	0.856

TESTING (4F: Img+Clinc): Primary (Canc Vs Norm) || Test (88 Vs 147)|| C2: Loc, CoOc& RunL

F1	Error	Sensitivity	Specificity	PPV	NPV	AUC
Comb1	0.209	0.568	0.925	0.82	0.782	0.853
Comb2	0.204	0.591	0.918	0.813	0.789	0.854
Comb4	0.191	0.614	0.925	0.831	0.8	0.877
Comb7	0.2	0.545	0.952	0.873	0.778	0.856
Comb9	0.221	0.591	0.891	0.765	0.784	0.856

III. Texture Features

**a. TRAINING (4 Features) : Primary: Cancer Vs Norm (78 Vs 166) || C3: Texture Features**							
**Features**	**Error**	**Sensitivity**	**Specificity**	**PPV**	**NPV**	**AUC**	
						**µ (σ)**	**CI**
F68:3D-Laws-10;F197:3D-WaveP2-L2-8;F214:3D-WaveP1-L2-25;F216:3D-WaveP1-L2-27	0.242	0.426	0.926	0.745	0.763	0.75(0.076)	[0.601,0.897]
F68:3D-Laws-10;F192:3D-WaveP2-L2-3;F215:3D-WaveP1-L2-26;F216:3D-WaveP1-L2-27	0.24	0.42	0.924	0.73	0.77	0.75(0.081)	[0.563,0.916]
F68:3D-Laws-10;F201:3D-WaveP2-L2-12;F214:3D-WaveP1-L2-25;F216:3D-WaveP1-L2-27	0.239	0.418	0.929	0.746	0.767	0.768(0.076)	[0.603,0.932]
F72:3D-Laws-14;F200:3D-WaveP2-L2-11;F215:3D-WaveP1-L2-26;F216:3D-WaveP1-L2-27	0.24	0.395	0.936	0.759	0.764	0.761(0.075)	[0.63,0.915]
F143:3D-Laws-94;F201:3D-WaveP2-L2-12;F214:3D-WaveP1-L2-25;F216:3D-WaveP1-L2-27	0.241	0.413	0.926	0.73	0.767	0.774(0.07)	[0.619,0.9]

**a. Clinc (4Feature): Primary (Canc Vs Norm) || Test (88 Vs 147)||**							
**Clinc**	**Error**	**Sensitivity**	**Specificity**	**PPV**	**NPV**	**AUC**	**CI**
Age, Gender, Pky, Smoke-Status	0.333	0.004	0.995	0.024	0.67	0.52 (0.066)	[0.377,0.65]

**a. ImageF+Clinc (4Feature): Primary (Canc Vs Norm) || Test (88 Vs 147)|| C3: Texture Features**							
**F1**	**Error**	**Sensitivity**	**Specificity**	**PPV**	**NPV**	**AUC**	**CI**
Comb1	0.242	0.426	0.914	0.694	0.775	0.757(0.068)	[0.601,0.883]
Comb2	0.252	0.409	0.919	0.721	0.757	0.758(0.071)	[0.604,0.868]
Comb4	0.249	0.392	0.923	0.708	0.763	0.763(0.069)	[0.62,0.914]
Comb5	0.243	0.382	0.934	0.741	0.761	0.749(0.081)	[0.535,0.886]
Comb8	0.25	0.408	0.916	0.711	0.765	0.763(0.067)	[0.613,0.9]

a. TESTING (4Feature): Primary (Canc Vs Norm) || Test (88 Vs 147 || C3: Texture Features (Sort: AUC, Sen/Spec)						
**Features**	**Error**	**Sensitivity**	**Specificity**	**PPV**	**NPV**	**AUC**
F68:3D-Laws-10;F197:3D-WaveP2-L2-8;F214:3D-WaveP1-L2-25;F216:3D-WaveP1-L2-27	0.243	0.477	0.925	0.792	0.747	0.824
F68:3D-Laws-10;F192:3D-WaveP2-L2-3;F215:3D-WaveP1-L2-26;F216:3D-WaveP1-L2-27	0.255	0.443	0.925	0.78	0.735	0.803
F68:3D-Laws-10;F201:3D-WaveP2-L2-12;F214:3D-WaveP1-L2-25;F216:3D-WaveP1-L2-27	0.255	0.443	0.925	0.78	0.735	0.82
F72:3D-Laws-14;F200:3D-WaveP2-L2-11;F215:3D-WaveP1-L2-26;F216:3D-WaveP1-L2-27	0.268	0.398	0.932	0.778	0.721	0.792
F143:3D-Laws-94;F201:3D-WaveP2-L2-12;F214:3D-WaveP1-L2-25;F216:3D-WaveP1-L2-27	0.243	0.5	0.912	0.772	0.753	0.819

e.TESTING (4F: Img+Clinc): Primary (Canc Vs Norm) || T12 Test (88 Vs 147)|| C3: Texture Features						
**F1**	**Error**	**Sensitivity**	**Specificity**	**PPV**	**NPV**	**AUC**
Comb1	0.243	0.455	0.939	0.816	0.742	0.804
Comb2	0.277	0.398	0.918	0.745	0.718	0.791
Comb4	0.285	0.375	0.918	0.733	0.711	0.806
Comb5	0.285	0.352	0.932	0.756	0.706	0.78
Comb8	0.268	0.42	0.918	0.755	0.726	0.807

Supplemental Table S10. Top images features discriminating cancer to benign nodules using 129 reproducible, non-redundant features. Features across all categories

Size Range:R1(4-12mm) & All Feature Categories: Youden metric (Sen/Spec, AUC)

**TRAINING (4 Feature) : Primary: Cancer Vs Norm (78 Vs 166) || All - R1 (4-12mm)**							
**Features**	**Error**	**Sensitivity**	**Specificity**	**PPV**	**NPV**	**AUC**	
						**μ (σ)**	**CI**
F17:9f-3D-Min-Dist-COG-to-Border;F26:EllipticFit;F34:Volume-pxl;F189:Hist-Skew-L1	0.155	0.335	0.974	0.737	0.857	0.683 (0.116)	[0.409,0.909]
F3:ShortAx;F26:EllipticFit;F34:Volume-pxl;F189:Hist-Skew-L1	0.153	0.33	0.975	0.745	0.857	0.663 (0.115)	[0.406,0.903]
F4:Mn-Hu;F26:EllipticFit;F34:Volume-pxl;F172:3D-Laws-123	0.16	0.313	0.966	0.691	0.858	0.754 (0.131)	[0.389,0.962]
F4:Mn-Hu;F26:EllipticFit;F34:Volume-pxl;F138:3D-Laws-89	0.149	0.294	0.983	0.753	0.856	0.748 (0.11)	[0.468,0.948]
F5:Std-Hu;F26:EllipticFit;F34:Volume-pxl;F189:Hist-Skew-L1	0.159	0.305	0.967	0.689	0.858	0.67 (0.099)	[0.452,0.882]

**Clinc (4Feature): Primary (Canc Vs Norm) || Test (88 Vs 147)||**							
**Clinc**	**Error**	**Sensitivity**	**Specificity**	**PPV**	**NPV**	**AUC**	**CI**
Age, Gender, Pky, Smoke-Status	0.191	0	0.999	0	0.809	0.615 (0.103)	[0.382,0.836]

**TRAIN: ImageF+Clinc (4Feature): Primary (Canc Vs Norm) || Test (88 Vs 147)|| R1: All**							
**F1**	**Error**	**Sensitivity**	**Specificity**	**PPV**	**NPV**	**AUC**	**CI**
Comb2	0.186	0.1	0.97	0.343	0.832	0.692(0.11)	[0.46,0.917]
Comb3	0.201	0.054	0.965	0.162	0.822	0.67(0.107)	[0.413,0.907]
Comb7	0.169	0.206	0.967	0.532	0.85	0.74(0.111)	[0.459,0.972]
Comb8	0.182	0.141	0.971	0.46	0.834	0.73(0.106)	[0.477,0.96]
Comb9	0.205	0.073	0.96	0.243	0.821	0.684(0.111)	[0.401,0.876]

**TESTING (4Feature): Primary (Canc Vs Norm) || Test (88 Vs 147)|| All – R1**						
**F1**	**Error**	**Sensitivity**	**Specificity**	**PPV**	**NPV**	**AUC**
Comb2	0.173	0.161	0.985	0.714	0.832	0.771
Comb3	0.167	0.161	0.992	0.833	0.833	0.786
Comb7	0.179	0.194	0.969	0.6	0.836	0.795
Comb8	0.191	0.161	0.962	0.5	0.829	0.778
Comb9	0.167	0.161	0.992	0.833	0.833	0.814

**TESTING (4F: Img+Clinc): Primary (Canc Vs Norm) || Test (88 Vs 147)|| R1- All**						
**F1**	**Error**	**Sensitivity**	**Specificity**	**PPV**	**NPV**	**AUC**
Comb2	0.179	0.129	0.985	0.667	0.827	0.732
Comb3	0.167	0.129	1	1	0.829	0.745
Comb7	0.173	0.194	0.977	0.667	0.837	0.767
Comb8	0.191	0.161	0.962	0.5	0.829	0.754
Comb9	0.173	0.129	0.992	0.8	0.828	0.765

Size Range: R2: (>12 -30mm)

**TRAINING (4 Feature) : Primary at T12: Cancer Vs Norm (78 Vs 166) || All - R2 (>12 -30mm)**							
**Features**	**Error**	**Sensitivity**	**Specificity**	**PPV**	**NPV**	**AUC**	
						**μ (σ)**	**CI**
F44:AvgCooC-Constrast;F196:3D-WaveP2-L2-7;F199:3D-WaveP2-L2-10;F214:3D-WaveP1-L2-25	0.211	0.913	0.569	0.815	0.732	0.702 (0.192)	[0.24,0.96]
F17:9f-3D-Min-Dist-COG-to-Border;F44:AvgCooC-Constrast;F204:3D-WaveP2-L2-15;F214:3D-WaveP1-L2-25	0.207	0.925	0.518	0.818	0.719	0.707 (0.152)	[0.37,0.965]
F44:AvgCooC-Constrast;F55:AvgRP;F204:3D-WaveP2-L2-15;F214:3D-WaveP1-L2-25	0.217	0.914	0.505	0.816	0.678	0.692 (0.143)	[0.4,0.933]
F21:10c-3D-Av-Vol-AirSpaces;F38:Length-Pxl;F64:3D-Laws-6;F204:3D-WaveP2-L2-15	0.224	0.906	0.499	0.81	0.68	0.684 (0.185)	[0.208,0.958]
F43:AvgCooc-Mp;F55:AvgRP;F204:3D-WaveP2-L2-15;F214:3D-WaveP1-L2-25	0.226	0.925	0.477	0.802	0.689	0.7 (0.181)	[0.288,0.963]

**Clinc (4Feature): Primary at T12 (Canc Vs Norm) || Prim at T12 Test (88 Vs 147)|| R2-(>12-30mm) All**							
**Clinc**	**Error**	**Sensitivity**	**Specificity**	**PPV**	**NPV**	**AUC**	**CI**
Age, Gender, Pky, Smoke-Status	0.388	0.92	0	0.651	0	0.36(0.162)	[0.068,0.645]

**TRAIN: ImageF+Clinc (4Feature): Primary at T12 (Canc Vs Norm) || Prim at T12 Test (88 Vs 147)|| R2 (>12-30mm): All**							
**F1**	**Error**	**Sensitivity**	**Specificity**	**PPV**	**NPV**	**AUC**	**CI**
Comb1	0.32	0.858	0.319	0.732	0.504	0.69(0.206)	[0.2,0.95]
Comb2	0.419	0.795	0.19	0.662	0.279	0.545(0.15)	[0.24,0.863]
Comb5	0.402	0.787	0.217	0.688	0.292	0.571(0.171)	[0.167,0.88]
Comb6	0.37	0.824	0.289	0.701	0.39	0.574(0.173)	[0.208,0.958]
Comb10	0.392	0.8	0.268	0.686	0.336	0.576(0.164)	[0.288,0.921]

**TESTING (4Feature): Primary at T12 (Canc Vs Norm) || Prim at T12 Test (88 Vs 147)|| All – R2**						
**F1**	**Error**	**Sensitivity**	**Specificity**	**PPV**	**NPV**	**AUC**
Comb1	0.315	0.739	0.375	0.872	0.2	0.601
Comb2	0.259	0.848	0.125	0.848	0.125	0.579
Comb5	0.259	0.848	0.125	0.848	0.125	0.606
Comb6	0.167	0.957	0.125	0.863	0.333	0.628
Comb10	0.222	0.87	0.25	0.87	0.25	0.614

**TESTING (4F: Img+Clinc): Primary at T12 (Canc Vs Norm) || Prim at T12 Test (88 Vs 147)|| R2 (>12-30mm)- All**						
**F1**	**Error**	**Sensitivity**	**Specificity**	**PPV**	**NPV**	**AUC**
Comb1	0.296	0.761	0.375	0.875	0.214	0.649
Comb2	0.259	0.848	0.125	0.848	0.125	0.571
Comb5	0.241	0.87	0.125	0.851	0.143	0.59
Comb6	0.167	0.935	0.25	0.878	0.4	0.723
Comb10	0.185	0.891	0.375	0.891	0.375	0.644

Supplemental Table S11. Top images features discriminating cancer to benign nodules in Indeterminate size range (R1: 4 to 12mm) using 129 reproducible, non-redundant features in three categories (Size & Shape, CooCc & Run. Length, Texture).

A) Range R1 (4 to 12mm) and C1: Size & Shape: Youden metric (Sen/Spec, AUC)

TRAINING (4 Feature) : Primary at T12: Cancer Vs Norm (78 Vs 166) || C1 & R1 (4 -12mm)							
Features	error	sensitivity	Specificity	PPV	NPV	AUC	
						µ (σ)	CI
F1:LongDia;F6:Vol-cm;F37:Thickness-Pxl;F14:9c-3D-Compact	.18	.115	0.989	0.517	0.824	0.556 (0.12)	[0.302,0.806]
F6:Vol-cm;F38:Length-Pxl;F40:Length-by-Width;F41:Border-Leng-Pxl	.168	.109	0.992	0.495	0.835	0.615 (0.107)	[0.415,0.811]
F6:Vol-cm;F38:Length-Pxl;F41:Border-Leng-Pxl;F30:Shape-Index	.178	.104	0.994	0.546	0.824	0.586(0.121)	[0.313,0.844]
F6:Vol-cm;F34:Volume-pxl;F38:Length-Pxl;F30:Shape-Index	.189	.114	0.982	0.471	0.82	0.619(0.111)	[0.333,0.822]
F36:Width-Pxl;F38:Length-Pxl;F40:Length-by-Width;F41:Border-Leng-Pxl	.173	.106	0.989	0.46	0.832	0.626(0.139)	[0.296,0.914]

Clinc (4Feature): Primary at T12 (Canc Vs Norm) || Prim at T12 Test (88 Vs 147)|| C1 & R1 (4 -12mm)							
Clinc	Error	Sensitivity	Specificity	PV	PV	AUC	CI
Age, Gender, Pky, Smoke-Status	0.191	0	0.999	0	0.81	0.57 (0.115)	[0.299,0.811]

TRAIN: ImageF+Clinc (4Feature): Primary at T12 (Canc Vs Norm) || Prim at T12 Test (88 Vs 147)|| C1 & R1 (4 -12mm)							
F1	Error	Sensitivity	Specificity	PV	PV	AUC	CI
Comb1	0.175	0.125	0.985	.517	.832	0.642(0.13)	[0.347,0.87]
Comb2	0.168	0.098	0.99	.393	.836	0.609(0.108)	[0.385,0.82]
Comb3	0.172	0.11	0.994	.535	.828	0.622(0.106)	[0.367,0.833]
Comb4	0 .177	0.129	0.984	.508	.83	0.629(0.112)	[0.349,0.806]
Comb5	0.171	0.122	0.989	.513	.833	0.647(0.125)	[0.374,0.89]

**TESTING (4Feature): Primary at T12 (Canc Vs Norm) || Prim at T12 Test (88 Vs 147)|| C1 & R1 (4 -12mm)**						
**F1**	**Error**	**Sensitivity**	**Specificity**	**PPV**	**NPV**	**AUC**
Comb1	0.154	0.258	0.985	0.8	0.849	0.679
Comb2	0.173	0.161	0.985	0.714	0.832	0.71
Comb3	0.173	0.194	0.977	0.667	0.837	0.697
Comb4	0.179	0.194	0.969	0.6	0.836	0.692
Comb5	0.198	0.032	0.985	0.333	0.811	0.671

**TESTING (4F: Img+Clinc): Primary at T12 (Canc Vs Norm) || Prim at T12 Test (88 Vs 147)|| C1 & R1 (4 -12mm)**						
**F1**	**Error**	**Sensitivity**	**Specificity**	**PPV**	**NPV**	**AUC**
Comb1	0.167	0.226	0.977	0.7	0.842	0.659
Comb2	0.167	0.161	0.992	0.833	0.833	0.667
Comb3	0.173	0.194	0.977	0.667	0.837	0.661
Comb4	0.167	0.194	0.985	0.75	0.838	0.659
Comb5	0.191	0.065	0.985	0.5	0.816	0.615

B) Range R1 (4 to 12mm): C2: Location, CoOc & RunL: Youden metric (Sen/Spec, AUC)

**TRAINING (4 Feature) : Primary at T12: Cancer Vs Norm (78 Vs 166) || C2: CoOc & RunL Features (Range: R1: 4-12mm).**							
**Features**	**Error**	**Sensitivity**	**Specificity**	**PPV**	**NPV**	**AUC**	
						**µ (σ)**	**CI**
F9:8b-3D-Bord-to-Lung;F17:9f-3D-Min-Dist-COG-to-Border;F19:10a-3D-Relat-Vol-Airspaces;F55:AvgRP	0.176	0.256	0.969	0.656	0.838	0.825 (0.087)	[0.633,0.972]
F9:8b-3D-Bord-to-Lung;F19:10a-3D-Relat-Vol-Airspaces;F5:Std-Hu;F48:AvgGLN	0.183	0.248	0.955	0.528	0.844	0.806 (0.091)	[0.598,0.965]
F9:8b-3D-Bord-to-Lung;F19:10a-3D-Relat-Vol-Airspaces;F5:Std-Hu;F55:AvgRP	0.183	0.252	0.949	0.507	0.847	0.801 (0.103)	[0.57,0.944]
F9:8b-3D-Bord-to-Lung;F19:10a-3D-Relat-Vol-Airspaces;F20:10b-3D-Num-AirSpaces;F55:AvgRP	0.189	0.246	0.951	0.499	0.839	0.768 (0.114)	[0.522,0.948]
F9:8b-3D-Bord-to-Lung;F17:9f-3D-Min-Dist-COG-to-Border;F48:AvgGLN;F52:AvgLRHGE	0.173	0.225	0.972	0.608	0.841	0.774 (0.103)	[0.461,0.932]

**Clinc (4Feature): Primary at T12 (Canc Vs Norm) || Prim at T12 Test (88 Vs 147)|| C2: RunG-CoOc -1 (4-12mm)**							
**Clinc**	**Error**	**Sensitivity**	**Specificity**	**PPV**	**NPV**	**AUC**	**CI**
Age, Gender, Pky, Smoke-Status	0.189	0	0.999	0	0.812	0.593 (0.127)	[0.342,0.878]

**TRAIN: ImageF+Clinc (4Feature): Primary at T12 (Canc Vs Norm) || Prim at T12 Test (88 Vs 147)|| C2: RunG-CoOc – R1 (4-12mm)**							
**F1**	**Error**	**Sensitivity**	**Specificity**	**PPV**	**NPV**	**AUC**	**CI**
Comb1	0.171	0.278	0.964	0.609	0.847	0.808(0.083)	[0.608,0.968]
Comb2	0.179	0.278	0.954	0.567	0.846	0.803(0.097)	[0.554,0.952]
Comb3	0.173	0.315	0.951	0.588	0.853	0.813(0.086)	[0.643,0.964]
Comb4	0.176	0.261	0.957	0.565	0.848	0.773(0.106)	[0.554,0.967]
Comb5	0.161	0.262	0.969	0.606	0.855	0.789(0.09)	[0.578,0.954]

**TESTING (4Feature: ImgF): Primary at T12 (Canc Vs Norm) || Prim at T12 Test (88 Vs 147 ||C2: RunG-CoOc Features, Range: R1 (4-12mm)**						
**Features**	**Error**	**Sensitivity**	**Specificity**	**PPV**	**NPV**	**AUC**
Comb1	0.167	0.226	0.977	0.7	0.842	0.784
Comb2	0.136	0.419	0.969	0.765	0.876	0.82
Comb3	0.148	0.387	0.962	0.706	0.869	0.831
Comb4	0.179	0.258	0.954	0.571	0.845	0.806
Comb5	0.179	0.194	0.969	0.6	0.836	0.73

**TESTING (4Feature: ImgF+Clinc): Primary at T12 (Canc Vs Norm) || Prim at T12 Test (88 Vs 147 || C2: RunG-CoOc, Range: R1 (4 - 12mm) .**						
**Features**	**Error**	**Sensitivity**	**Specificity**	**PPV**	**NPV**	**AUC**
Comb1	0.185	0.129	0.977	0.571	0.826	0.766
Comb2	0.148	0.323	0.977	0.769	0.859	0.823
Comb3	0.154	0.29	0.977	0.75	0.853	0.829
Comb4	0.179	0.226	0.962	0.583	0.84	0.803
Comb5	0.167	0.194	0.985	0.75	0.838	0.707

Background Information for Supplemental Figure SF1.

We also attempted to find stable features across non-growing controls over three screening scans that were acquired about a year apart. We observed large variations in features due to patient level scan differences across time. In this sub-study, 26 features were stable at a CCC ≥ 0.6; and these were included as part of the test-retest repeatable feature set (See *Supplemental Figure SF1*).

Supplemental Figure SF1. Differential variability plots (Bland-Altman plots) between non-cancerous across time points (NC1 & 2 are Non-cancer cohort 1 &2). A) Longest diameter for first cohort (top panel). B) Longest diameter for second cohort (bottom panel) C) Size distribution (longest diameter & volume) across time points.

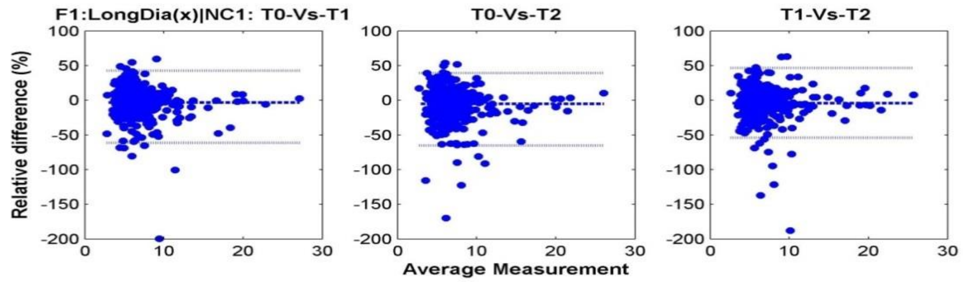

(a)

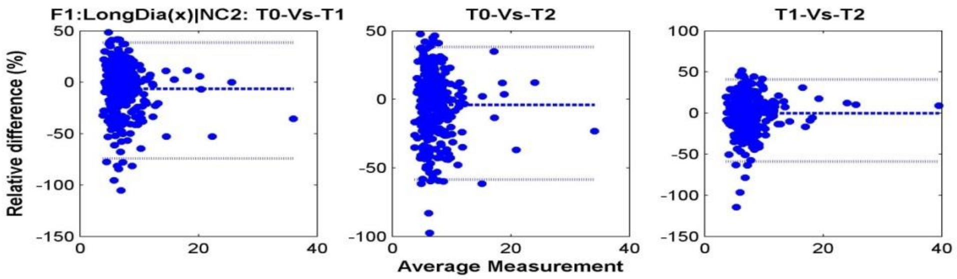

(b)

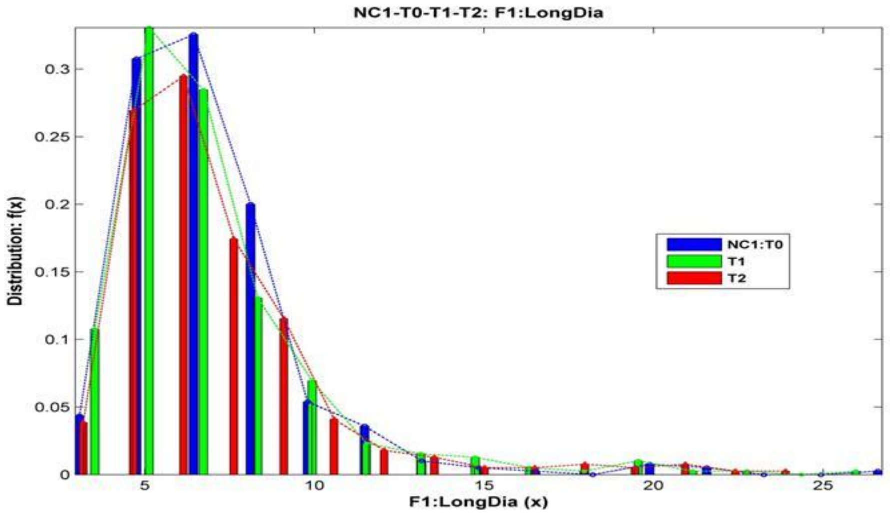
NC1-T0-T1-T2: F1:LongDia
Distribution: f(x)
F1:LongDia (x)
NC1:T0
T1
T2
0
0.05
0.1
0.15
0.2
0.25
0.3
5
10
15
20
25

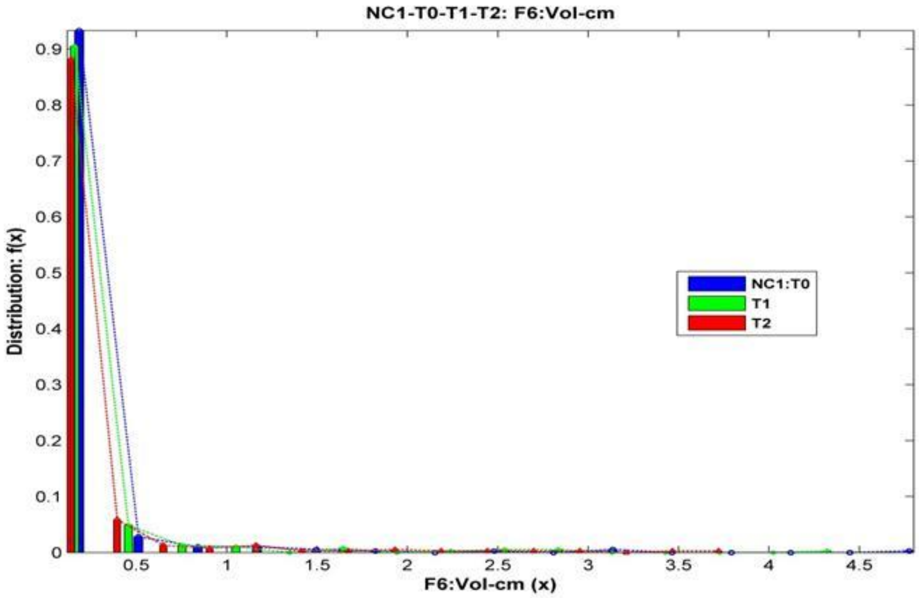
NC1-T0-T1-T2: F6:Vol-cm
Distribution: f(x)
F6:Vol-cm (x)
NC1:T0
T1
T2
0
0.1
0.2
0.3
0.4
0.5
0.6
0.7
0.8
0.9
0.5
1
1.5
2
2.5
3
3.5
4
4.5

(c)

**Concordance Correlation Coefficient:** Normal Cases (Nodule+, non-Cancer) **(T0 Vs T1, T0 Vs T2, T1 Vs T2) : Min**							
**Category**	**Feature Type**	**Subtotal**	**Percent of features (Num. Features)**				
			≤ 60%	≥ 70%	≥ 80%	≥ 85%	≥ 90%
C1.Size & Shape	Size	13	84.6 (11)	76.9 (10)	46.2(6)	0 (0)	0 (0)
	Shape	12	25 (3)	16.7(2)	0 (0)	0 (0)	0 (0)
C2. Location, RunL & CoOc, Histogram	Location	14	42.9 (6)	35.7(5)	35.7 (5)	14.3	0 (0)
	Histogram	8	37.5 (3)	12.5(1)	0 (0)	0 (0)	0 (0)
	Runlength & CoOcc	17	17.6 (3)	17.7(3)	11.7 (2)	0 (0)	0 (0)
C3. Texture: Laws&Wavelet	Laws	125	0.8 (1)	0.8 (1)	0 (0)	0 (0)	0 (0)
	Wavelet	30	0 (0)	0 (0)	0 (0)	0 (0)	0 (0)
Total		219	12.3 (27)	10.1 (22)	5.9 (13)	0.913 (2)	0 (0)

Supplemental Figure SF2. The figure shows the workflow to assess classifier performance due to reconstruction kernels. The classifier was formed using a data set with one type of kernel (in a manufacturer) to discriminate malignant lung nodules and tested on a data set with different kernel type used by the same manufacturer, keeping other parameters constant. The variability in the predictions (AUC) was evaluated and reported as percent change. The figures contrast visual difference in image reconstructions of two different convolution kernels (across columns), where each row represents a patient nodule The panel shows A) workflow for the reconstruction study B) Patient scans reconstructed using Siemens scanner (B30F, B50F, slice thickness 2mm) C) Patient scans resconstructed using General Electric (GE) scanner (Standard & Bone, slice thickness 2.5mm). Details of the analysis will be reported in our following study (11).

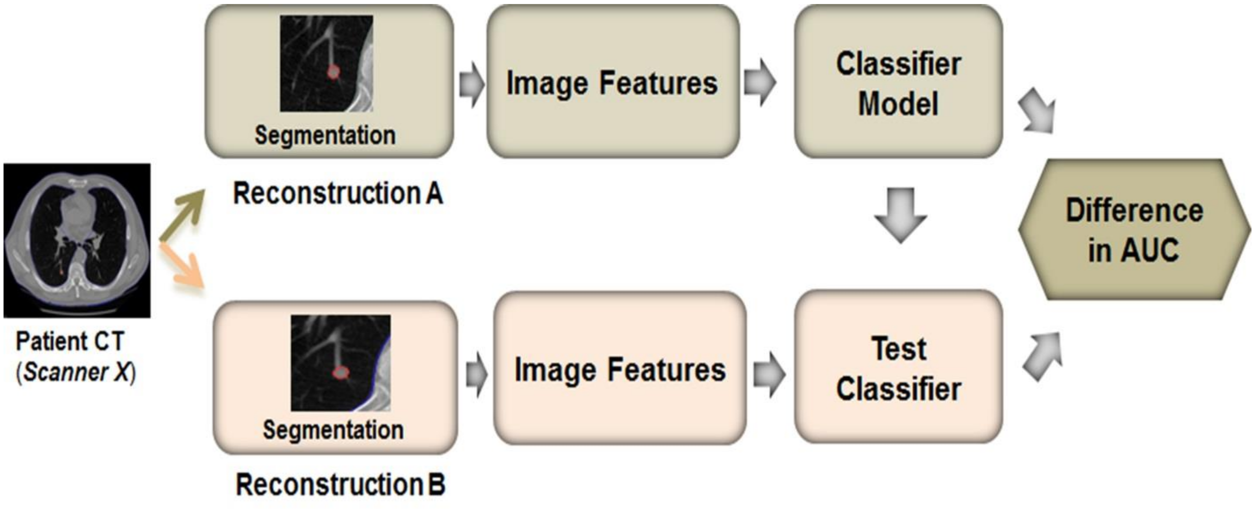

(A)

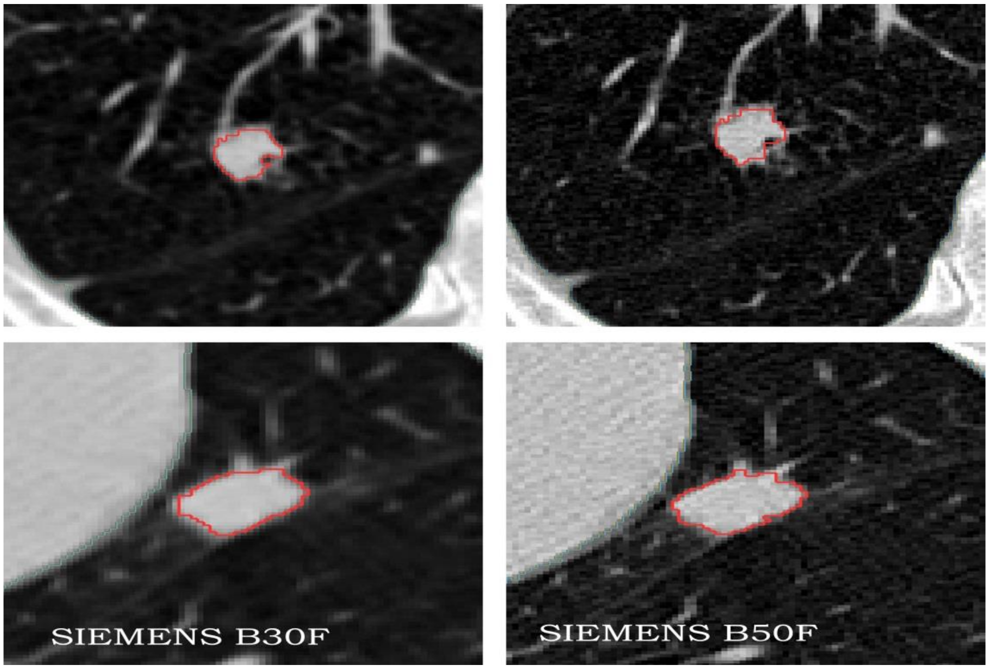

(B)

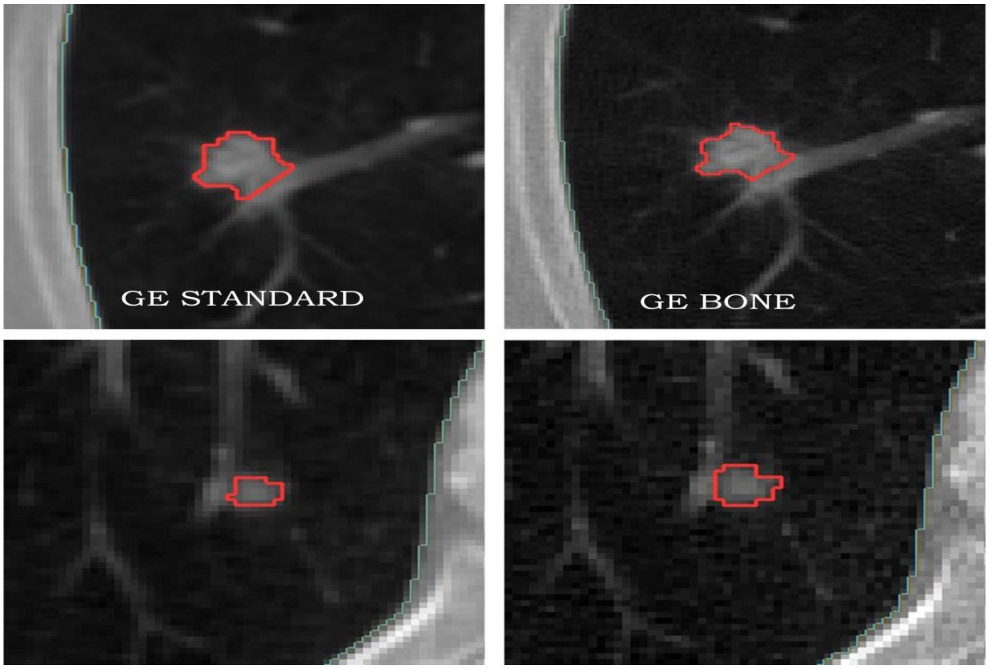

(C)

Supplemental Figure SF**3**. Size distribution of lung nodules used in the training and validation (test) cohorts A) Longest diameter (mm), B) Volume (cm).

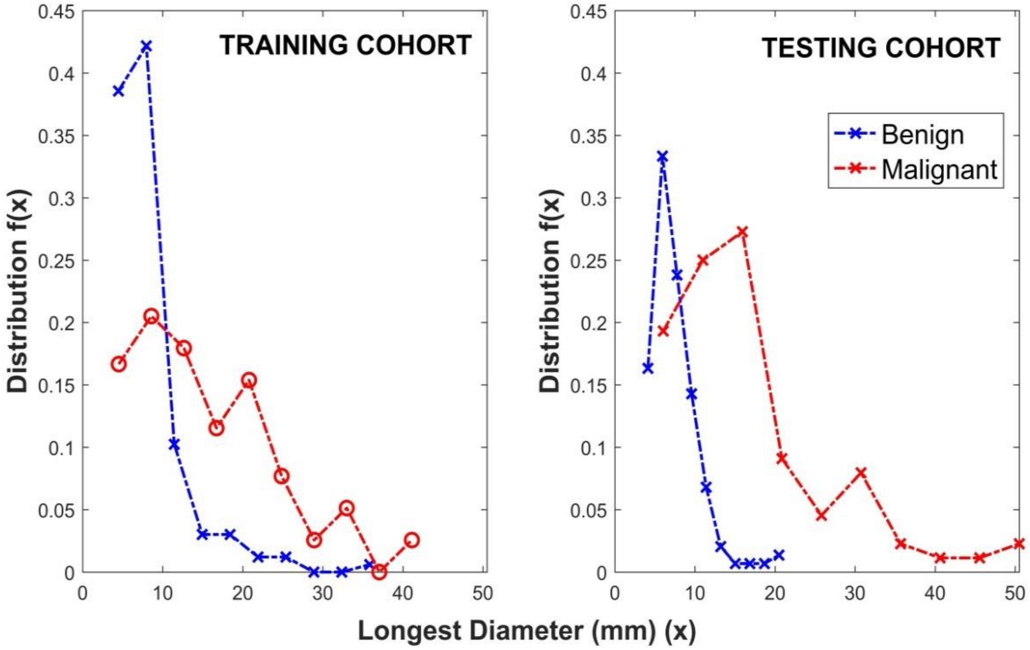

(A)

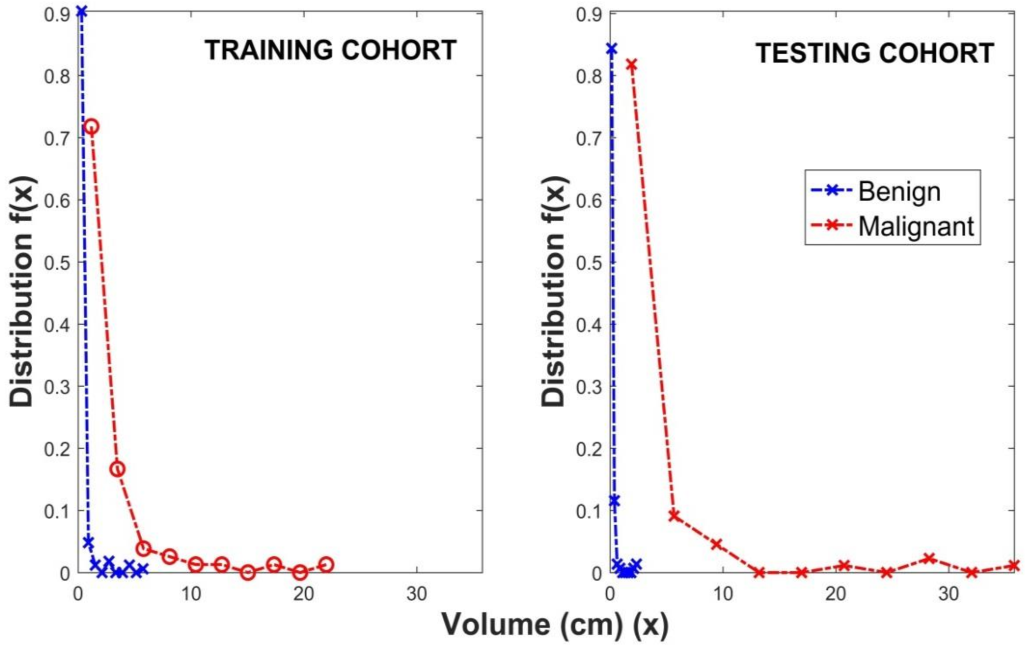

(B)

Reference

1. Schonmeyer R, Athelogou M, Sittek H, Ellenberg P, Feehan O, Schmidt G, Binnig G. Cognition Network Technology prototype of a CAD system for mammography to assist radiologists by finding similar cases in a reference database. International journal of computer assisted radiology and surgery. 2011;6(1):127-34. Epub 2010/05/27. doi: 10.1007/s11548-010-0486-8. PubMed PMID: 20503075.
2. Balagurunathan Y GY, Wang H, Kumar V, Grove O, Hawkins S, Kim J, Goldgof DB, Hall LO, Gatenby RA, Gillies RJ. Reproducibility and Prognosis of Quantitative features extracted from CT images Transl Oncol. 2014;7(1):72-87.
3. Xu D-H, Kuran AS, Furst JD, DS. R. RUN-LENGTH ENCODING FOR VOLUMETRIC TEXTURE. 4th IASTED Int'l Conf on Visualization, Imaging and Image Processing; Septermber 6-8; Marbella, Spain.2004.
4. Tang X. Texture information in run-length matrices. IEEE Transactions on Image Processing. 1998;7(11):1602-9.
5. A.S. Kurani D-HX, J.D. Furst, and D.S. Raicu. Co-occurrence matrices for volumetric data. 7th IASTED Int'l Conf on Computer Graphics and Imaging2004.
6. Mokji M.M ABSAR. Gray Level Co-Occurrence Matrix Computation Based On Haar Wavelet. IEEE Computer Graphics, Imaging and Visualisation (CGIV '07): IEEE Aug 2007. p. 273-9.
7. V.A. Kovalev FK, H.-J Gertz, and D.Y. von Cramon. Three-dimensional texture analysis of MRI brain datasets. IEEE Trans on Medical Imaging. 2001;20(5):424-33.
8. Laws K. Texture Image Segmentation. Los Angeles: University of South California 1980.
9. Benke K K CDaSDR. A study of the effect of image quality on texture energy measures. Meas Sci Technol. 1994;5:400-7.
10. K. Jafari-Khouzani HS-Z, K. Elisevich, and S. Patel. Comparison of 2D and 3D wavelet features for the lateralization. In Proc of SPIE Medical Imaging 2004: Physiology, Function and Structure from Medical Images. 2004;5369:593-601.
11. Balagurunathan Y, Moros E, Garcia A, Schabath M, Gillies RJ. Effects of CT scanner variability on pulmonary nodule discrimination. (in preparation). 2017.
